# Polycomb protein RYBP facilitates super-enhancer activity

**DOI:** 10.1186/s10020-024-01006-3

**Published:** 2024-11-27

**Authors:** Yu Hong, Ranran Dai, Xinlan Li, He Xu, Chao Wei

**Affiliations:** 1 Department of Pharmacy, The First Affiliated Hospital, Sun Yat-sen University, Guangzhou, 510080 China; 2 Zhongshan School of Medicine, Sun Yat-Sen University, Guangzhou, 510080 China; 3 Guangdong Provincial Key Laboratory of New Drug Design and Evaluation, School of Pharmaceutical Sciences, Sun Yat-sen University, Guangzhou, 510006 China; 4 Center of Translational Medicine, The First Affiliated Hospital, Zhongshan School of Medicine, Sun Yat-Sen University, Guangzhou, 510080 China

**Keywords:** Epigenetic, Polycomb complex, Enhancer, Stem cell, Histone modification

## Abstract

**Background:**

Polycomb proteins are conventionally known as global repressors in cell fate determination. However, recent observations have shown their involvement in transcriptional activation, the mechanisms of which need further investigation.

**Methods:**

Herein, multiple data from ChIP-seq, RNA-seq and HiChIP before or after RYBP depletion in embryonic stem cell (ESC), epidermal progenitor (EPC) and mesodermal cell (MEC) were analyzed.

**Results:**

We found that Polycomb protein RYBP occupies super-enhancer (SE) in ESCs, where core Polycomb group (PcG) components such as RING1B and EZH2 are minimally enriched. Depletion of RYBP results in impaired deposition of H3K27ac, decreased expression of SE-associated genes, and reducing the transcription of enhancer RNA at SE regions (seRNA). Regarding the mechanism of seRNA transcription, the Trithorax group (TrxG) component WDR5 co-localizes with RYBP at SEs, and is required for seRNA expression. RYBP depletion reduces WDR5 deposition at SE regions. In addition, TrxG-associated H3K4me3 tends to be enriched at SEs with high levels of seRNA transcription, and RYBP deficiency impairs the deposition of H3K4me3 at SEs. Structurally, RYBP is involved in both intra- and inter-SE interactions. Finally, RYBP generally localizes at SEs in both in vitro cell lines and in vivo tissue-derived cells, dysfunction of RYBP is associated with various cancers and developmental diseases.

**Conclusion:**

RYBP cooperates with TrxG component to regulate SE activity. Dysfunction of RYBP relates to various diseases. The findings provide new insights into the transcriptionally active function of Polycomb protein in cell fate determination.

**Supplementary Information:**

The online version contains supplementary material available at 10.1186/s10020-024-01006-3.

## Background

The discovery of Polycomb group proteins unveiled the existence of global repressors (Lewis 1978), and has been found to be crucial for controlling segmentation in *Drosophila* (McKeon et al. 1994). Dysregulation of PcG proteins is associated with various developmental abnormalities and cancers, including embryonic lethality, colorectal cancer and bladder tumors, prostate cancer, and breast cancer (Bachmann et al. 2006; Mimori et al. 2005; Pasini et al. 2004; Weikert et al. 2005). PcG family comprises two main subfamilies: Polycomb Repressive Complex 1 (PRC1) and Polycomb Repressive Complex 2 (PRC2) (Gao et al. 2012; Hisanaga et al. 2023; Meng et al. 2020). Core components of PRC2, including EED, SUZ12, EZH1, and EZH2, catalyze histone H3 lysine 27 trimethylation (H3K27me3) (Hisanaga et al. 2023; Meng et al. 2020). PRC1 mediates mono-ubiquitylation of histone H2A at position 119 (H2AK119ub1) and consists of several members such as CBX (CBX2, CBX4, CBX6, CBX7, CBX8), PCGF (PCGF1, PCGF2, PCGF3, PCGF4, PCGF5, PCGF6), PHC (PHC1, PHC2, and PHC3), RING1A, RING1B, RYBP or YAF2 (Gao et al. 2012; Morey et al. 2013; Tavares et al. 2012). Based on the presence or absence of CBX7 and RYBP, PRC1 can be further categorized into canonical PRC1 (CBX7-defined) and non-canonical PRC1 (RYBP-defined) (Morey et al. 2013; Tavares et al. 2012). Canonical PRC1 and non-canonical PRC1 can cooperatively regulate the suppression of developmental genes, while also independently regulating specific sets of genes (Morey et al. 2012). Crosstalk between PRC1 and PRC2 collectively mediates developmental gene repression (Eskeland et al. 2010; Francis et al. 2004; Morey et al. 2012; Pengelly et al. 2015; Tavares et al. 2012). Typically, PRC2 catalyzes H3K27me3, which is recognized by canonical PRC1 to catalyze H2AK119ub1, leading to chromatin compaction and silencing of lineage commitment genes (Eskeland et al. 2010; Francis et al. 2004; Morey et al. 2012; Pengelly et al. 2015; Tavares et al. 2012). On the contrary, the deposition of RYBP-defined non-canonical PRC1 is independent of H3K27me3 (Tavares et al. 2012). During this process, non-canonical PRC1 firstly catalyzes H2AK119ub1, recruiting PRC2 and forms the Polycomb domain (Blackledge et al. 2014).

A previous study indicated that a subset of genes occupied by RYBP exhibits higher transcription levels compared to those bound by CBX7 (Morey et al. 2013). These RYBP-targeted genes are enriched in cellular maintenance terms such as metabolism and cell-cycle progression (Morey et al. 2013), suggesting a transcriptional activation function of Polycomb protein. In differentiated cells, the PRC2 component EZH1 is required for RNA polymerase II elongation (Mousavi et al. 2012). The mechanism underlying how PcG components activate gene expression needs further elucidation.

In the present work, it was observed that RYBP localizes to SE regions with minimal deposition of other PcG components. Loss of RYBP led to impaired SE activity, characterized by reduced deposition of H3K27ac and diminished expression of enhancer RNAs (eRNAs) at SEs. Furthermore, RYBP was found to cooperate with TrxG components to promote H3K4me3 deposition at SEs, a modification associated with eRNA transcription. Lastly, dysregulation of RYBP was linked to various disease pathologies.

## Materials and methods

### Cell lines and culture conditions

Wild-type (WT) mouse ESCs were maintained in ESC medium composed of DMEM (Hyclone, SH30022.01), 15% fetal bovine serum (ThermoFisher, 10099141 C), and 1000 U/mL recombinant LIF (Millipore, ESG1107), 0.1 mM β-mercaptoethanol (ThermoFisher, 31350010), 100× Glutamax (ThermoFisher, 35050061), 100× nonessential amino acids (ThermoFisher, 11140050). *Rybp*-floxed ESCs were cultured in ESC medium, RYBP-depleted ESCs were generated by supplementing the medium with 5 µM 4-hydroxytamoxifen (4-OHT, Sigma, H7904) for 4 days.

Data of embryonic development in Fig. 5H was obtained from public data set (GSE22182), and the detailed methods were described in the published literature (Tang et al. 2011). In brief, at E1.5, E2.0, and E2.5, two-cell, four-cell, and eight-cell embryos were flushed from the oviduct. Subsequently, individual blastomeres were separated by gently pipetting with a glass capillary in calcium-free medium. The isolated single cells were then washed twice in 0.1% BSA before being individually picked for further analysis.

### Differentiation of ESCs to mesodermal cells

The *Rybp*-floxed ESCs were dissociated into single cells and seeded at a density of 200,000 cells per well in low-attachment six-well plates. The cells were cultured in M1 medium, which consists of a 3:1 ratio of IMDM (Hyclone, SH30228.02) to F12 (ThermoFisher, 11765054), supplemented with 0.05% BSA (Sigma, A7906), 100× N2 (Gibco, 17502048), 50× B27 (Gibco, 12587010), 450 µM MTG (Sigma, M6145), and 50 µg/mL vitamin C (Merck, A4544). After 48 h, the EBs were dissociated into single cells and reseeded onto low-attachment plates, and were cultured in MEC medium, which consists of M1 medium and 5 ng/mL VEGF (Peprotech, 450 − 32), 8 ng/mL Activin A (Peprotech, 120-14E), and 1 ng/mL BMP4 (Peprotech, 315 − 27). The cells continued to be cultured for an additional 40 h. Partial embryoid bodies (EBs) were then cultured in MEC medium with or without 4-OHT for 4 days. These EBs were used for RNA extraction to assess gene expression. Additionally, partial EBs were dissociated into single cells and replanted into 96-well plates in MEC medium with or without 4-OHT for another 4 days. Subsequently, cell viability was assessed using the CCK-8 (Dojindo, CK04) assay to determine cell numbers.

### Differentiation of stem cells to cardiomyocytes

The differentiation experiment in Fig.S5B was performed according to the established protocol (Lin et al. 2017). 70–80% confluent hESCs were transferred to differentiation medium, which consisted of DMEM/F12 (C11330500BT, Gibco) supplemented with 10.7 µg/ml transferrin (T0665, Sigma), 71 µg/ml L-ascorbic acid-2-phosphate magnesium (A8960, Sigma), 14 ng/ml sodium selenite (S5261, Sigma), and 1× Chemical Defined Lipid Concentrate (11905031, Gibco). This day was designated as Day 0. From Day 0 to Day 1, the medium was supplemented with 6 µM CHIR99021 (S1263, Selleck). On Day 2, the cells were transitioned to medium 1 with the addition of 3 µg/ml Heparin (S1346, Selleck) until Day 7. Additionally, from Day 2 to Day 5, the culture medium was supplemented with 3 µM IWP2 (S7085, Selleck). Starting from Day 7, the hESC-derived cardiomyocytes were cultured in differentiation medium 1, and supplemented with 20 µg/ml insulin (91077 C, Sigma). Typically, spontaneous contractile clusters of human cardiomyocytes (hCMs) emerged by Day 7.

The differentiation data in Fig. 5I were obtained from public data (GSE145623), and the detailed methods was described in a patent (Biomaterials for 3D Cell Growth and Differentiation). In brief, mouse ESCs were seeded into plates coated with crosslinked elastin-like proteins. They were initially cultured in LIF-free ES culture medium for 2 days. After this period, the medium was replaced with GMEM supplemented with 2.5% KnockOut Serum Replacement, 1% NEAA, and 10 µM β-mercaptoethanol for the following 12 days.

### Reprogramming of MEF to iPSCs

The reprogramming experiment in Fig.S5C was performed according to an established protocol (Chen et al. 2011). In detail, retroviral vectors containing the cDNAs of *Oct4*, *Sox2*, and *Klf4* were transfected into PlatE cells using calcium phosphate transfection to generate the viruses. Mouse embryonic fibroblasts (MEFs) within one passage were seeded in dishes at a density of 5000 cells/cm² and cultured in MEF medium (DMEM with 10% FBS) for 24 h. Subsequently, the medium was replaced with MEF medium containing the *Oct4*,* Sox2*, and *Klf4* viruses and 4 µg/ml polybrene (Sigma, H9268). After 24 h, the medium was refreshed with new MEF medium containing the virus and 4 µg/ml polybrene for an additional 24 h. Following this infection period, the medium was changed to iCD1 medium (Chen et al. 2011), and the cells were cultured for 7 days to generate induced pluripotent stem cells (iPSCs).

Reprogramming data in Fig. 5J were obtained from public data (GSE19023), and the detailed methods was described in the published literature (Heng et al. 2010). In brief, MEFs were infected with viruses carrying the reprogramming factors. The medium was refreshed with new MEF medium one day post-infection. On the second day after infection, the cells were transferred to MEF feeder layers and maintained in FBS-containing medium for 6 days. Subsequently, the cells were cultured for an additional 5 to 15 days in medium supplemented with KnockOut Serum Replacement.

### Embryoid body (EB) formation

WT and RYBP knockout (KO) mouse ESCs were obtained from the Li laboratory (Zhao et al. 2020). These cells were cultured in low-adhesion dishes in ESC medium lacking LIF. EBs were collected on Days 2, 4, 6, 8, 10, and 14, and RNA was extracted from the formed EBs.

### RNA extraction

The extraction of RNA was performed according to the instructions of the commercial RNA extraction kit (Promega, LS1040). First, 3 to 5 million cells were resuspended in 0.5 ml of RNA lysis buffer, allowing sufficient time for complete cell lysis. Then, an equal volume of RNA dilution buffer was added and mixed thoroughly. The mixture was centrifuged at 13,000 g for 5 min at 4 °C. The supernatant was carefully transferred to a new tube. Subsequently, 0.5 volumes of ethanol were added to the supernatant and mixed thoroughly. This mixture was transferred to an RNA-binding column to allow RNA adsorption. The column was washed once with wash buffer. Following this, the column was treated with DNase I at room temperature for 15 min to degrade any contaminating DNA. The column was then washed twice with wash buffer. Finally, the purified RNA was eluted with RNase-free water.

### Co-immunoprecipitation (Co-IP)

Biotin-tagged WDR5 ESC pellets were suspended in nuclear extract buffer A, which consists of 10 mM HEPES (GIBCO, 15630-080), 1.5 mM MgCl2 (Merck, M4880), 10 mM KCl (Merck, 60142), 0.5 mM DTT (Merck, 646563), 0.2 mM PMSF (Merck, 78830), and protease inhibitor cocktail (Merck, P8340). After centrifugation and washing with nuclear extract buffer A, the cell pellets were resuspended in nuclear extract buffer C, which consists of 20 mM HEPES (GIBCO, 15630-080), 20% glycerol (Merck, G6279), 0.42 M NaCl (Merck, S5150), 1.5 mM MgCl2 (Merck, M4880), 0.2 mM EDTA (ThermoFisher, AM9260G), 0.5 mM DTT (Merck, 646563), 0.2 mM PMSF (Merck, 78830), and protease inhibitor cocktail (Merck, P8340) and rotated at 4 °C for 2 h. The insoluble material was removed by centrifugation at 20,000 g at 4 °C for 30 min. The supernatant was then diluted 5-fold with buffer DNP, which consists of 20 mM HEPES (GIBCO, 15630-080), 20% glycerol (Merck, G6279), 100 mM KCl (Merck, 60142), 1.5 mM MgCl2 (Merck, M4880), 0.2 mM EDTA (ThermoFisher, AM9260G), 0.02% NP40 (Sangon, A100109), 0.5 mM DTT (Merck, 646563), 0.2 mM PMSF (Merck, 78830), and protease inhibitor cocktail (Merck, P8340) and incubated with 4 ug RYBP (Abcam, ab5976) antibody or IgG (Merck, PP64) antibody-coated protein G-agarose. After three washes with buffer DNP, the samples on beads were eluted by 1.5X SDS sample buffer for western blot analysis.

### Western blot

Total proteins were extracted from cultured cells using CytoBuster (Merck, 71009-M) at 4 °C for 15 min. Then, the proteins were separated on 12% sodium dodecyl sulfate–polyacrylamide gel electrophoresis (SDS-PAGE) gels and transferred to a 0.45-µm PVDF membrane (BIO-RAD, 1620177) via wet transfer. The membrane was then blocked with 5% BSA in TBST for 1 h at room temperature. Following this, the membrane was incubated with primary antibodies (RYBP, 1:1000, santa cruz, sc374235), WDR5 (1:500, santa cruz, sc-393080), SA-HRP (1:5000, YEASEN, 35105ES60), H3K4me3 (1:10000, ABclonal, A22146) overnight at 4 °C. After three washes for 10 min each, the membrane was incubated with Anti-rabbit IgG (1:1000, CST 7074P2) or Anti-mouse IgG (1:1000, CST, 7076P2) for 1 h at room temperature. Finally, the membranes were treated with Femto ECL substrate (BIO-RAD, 1705061) and the band signals were visualized.

### ChIP-seq

ChIP-seq experiments were conducted following procedures described in a previously published study (Lee et al. 2006). The antibodies used were 5 µg anti-RYBP (Abcam, ab5976), 5 µg anti-WDR5 (Bethyl Laboratories, A302-429 A), 2 µg anti-H3K4me3 (Abcam, ab8580), and 2 µg anti-H3K27ac (Abcam, ab4729). These antibodies were incubated with Dynabeads (ThermoFisher, 10004D) at 4 °C overnight. Cells (5 × 10⁷ for RYBP and WDR5 antibodies, 1 × 10⁷ for H3K4me3 and H3K27ac antibodies) were cross-linked with formaldehyde (Sigma, F8775) for 10 min. The cross-linking was quenched by adding 2.5 M glycine (Solarbio, G8200). The cells were then sonicated to fragment the DNA to sizes between 200 and 500 bp. The chromatin complexes were subjected to immunoprecipitation using the antibody-coated beads at 4 °C overnight. DNA-bound beads were washed four times to remove unbound DNA. The DNA was eluted with 210 µl of elution buffer. Cross-links were reversed by incubating the samples at 65 °C overnight. The samples were treated with RNase A (ThermoFisher, EN0531) at 37 °C for 2 h to remove RNA, followed by treatment with Proteinase K (ThermoFisher, 25530049) to eliminate proteins. DNA purification was achieved by adding a phenol: chloroform: isoamyl alcohol mixture (Amresco, 0883), followed by precipitation with ethanol. Finally, DNA pellet was then dissolved in of H₂O for subsequent sequencing. Two replicates were used for each group.

### HiChIP

HiChIP experiments were conducted following a well-established public method (Mumbach et al. 2016) with slight modifications. A total of 15 million mESCs were cross-linked with formaldehyde (Sigma, F8775) for 10 min. The cross-linking reaction was quenched by adding of 2.5 M glycine (Solarbio, G8200). The cells were subsequently resuspended in Hi-C Lysis Buffer and centrifuged at 2500 rcf for 5 min, after which the supernatant was discarded. The resulting cell pellets were digested at 37 °C for 2 h using 375 U of MboI restriction enzyme (NEB, R0147). Following digestion, proximity ligation was performed by adding a mixture comprising 37.5 µL of biotin-dATP (ThermoFisher, 19524016), 1.5 µL of 10 mM dCTP (ThermoFisher, 18253013), 1.5 µL of 10 mM dGTP (ThermoFisher, 18254011), 1.5 µL of 10 mM dTTP (ThermoFisher, 18255018), and 10 µL of DNA Polymerase I (NEB, M0210). This mixture was incubated at 37 °C for 1 h with gentle rotation. Subsequently, a 948 µL ligation master mix containing 3 µL 50 mg/mL BSA (sigma, A7906), and 10 µL 400 U/µL T4 DNA Ligase (NEB, M0202) was added, and the mixture was incubated at room temperature for 4 h with rotation. After centrifugation of the nuclei at 2500 rcf for 5 min, the supernatant was discarded. 6 µL of lambda exonuclease (NEB, M0262L) and 6 µL of exonuclease I (NEB, M0293L) were used to further treat the sample at 37 °C for 1 h. Following this incubation, a centrifugation step was performed at 2500 rcf for 5 min at 4 °C. The resulting pellet was resuspended in 880 µL of nuclear lysis buffer and sheared to generate DNA fragments approximately 500–1000 bp in size. Subsequently, 90 µL of Protein A (ThermoFisher, 10002D) beads were added to the sample and rotated at 4 °C for 1 h. The sample was then placed on a magnet, and the supernatant was transferred to new tubes. Following this, the sample was incubated overnight at 4 °C with rotation after the addition of 12 µg of anti-RYBP antibody (Abcam, ab5976). An additional 90 µL of Protein A beads were introduced to the sample, which were then rotated at 4 °C for 4 h. After performing three washes of the beads, the sample was resuspended in 100 µL of DNA Elution Buffer to elute the DNA. Each sample received 10 µg of Proteinase K (Invitrogen, 100005393) and was incubated at 55 °C for 45 min with shaking. DNA purification was achieved by adding a phenol: chloroform: isoamyl alcohol mixture (Amresco, 0883), followed by precipitation with ethanol. The resulting DNA pellet was then dissolved in 21 µL of H₂O. Finally, biotinylated DNA was captured using Streptavidin C-1 beads (ThermoFisher, 65001), and a commercial library preparation kit (Vazyme, TD502) was employed for library construction.

### ChIP-seq data analysis

Trim_galore (version 0.6.6) (Fóthi et al. 2024) was used to assess the quality of FASTQ files (parameters: trim_galore.py -i ./rawdata/sample.fq.gz). Then `Bowtie2` (v2.3.5.1)(Langmead and Salzberg 2012) with end-to-end alignment was used to align the trimmed reads to the mm9 or Rnor_6.0 reference (parameters: bowtie2.py -x ./Genome -i ./Reads/ sample.fq.gz -o ./). Subsequently, PCR duplicates from the resulting BAM files were removed using samtools sorted, and indexed with `Samtools` (v1.3.1) (Li et al. 2009) (parameters: samtools rmdup -s sample.bam ./ sample.sorted.bam). Index was established via `Samtools` (parameters: samtools index sample.bam). DeepTools (v3.3.0) (Ramírez et al. 2014) bamCoverage was used to generate bigwig files (parameters: bamCoverage -b sample.bam --normalizeUsing RPKM -p max -o sample.bw). Peaks were identified using MACS2 (v2.1.1) (Zhang et al. 2008) with a false discovery rate (FDR) threshold of 0.01, comparing treatment and input samples to detect significant binding sites. Peaks were identified if their false discovery rate (FDR) was below 0.01.

To incorporate replicates into the analysis, BAM files from biological replicates were merged using samtools merge. DeepTools computeMatrix and plotHeatmap functions were used to create heatmaps for visualization, the detailed codes could be observed in Supplementary Material 3. Data of quality control was provided in Table S1.

### HiChIP-seq data analysis

The initial trimming of adapters was conducted using fastp (version 0.20.0) (Chen et al. 2018) with the following parameters: `fastp --thread 8 − 5 -3 -i rawdata/sample_1.fastq.gz -o QC/sample_1.fastq -I rawdata/sample_2.fastq.gz -O QC/sample_2.fastq`. Subsequent mapping, filtering, correction, and binning of HiChIP data were carried out utilizing HiC-Pro (version 2.11.1) (Servant et al. 2015), with parameters set as follows: `HiC-Pro -c config.txt -i QC -o hicpro_outfile`. The aligned paired-end reads, which had undergone adapter trimming, were mapped to the mm9 reference genome. Following this, HiC-Pro was employed to filter out self-circle ligation, dangling ends, re-ligation, and other unwanted types after the mapping process. Raw contact matrices were generated at various resolutions: 10 kb, 25 kb, 50 kb, 100 kb, 500 kb, and 1 Mb. For the correction of raw contact matrices, the iterative correction method (ICE) was applied. To convert the allValidPairs output from the pipeline into Juicebox hic format at fragment resolution, the hicpro2juicebox.sh utility was utilized. The conversion of ValidPairs from the HiC-Pro results for all replicates into BEDPE format was accomplished using the hicpro2bedpe.py script, with the following parameters: `hicpro2bedpe.py -f sample_merged.allValidPairs -o hic2bedpe`.

Chromatin loops were identified with the cLoops tool (Cao et al. 2020) (version 0.93a, parameters: `cLoops -f hic2bedpe/sample_merged.bedpe.gz -o cLoops/ sample_merged -w -j -s -eps 1000,2000,5000 -minPts 10 -p 16`).

### RNA-seq data analysis

Trim_galore (version 0.6.6) (Fóthi et al. 2024) was used to assess the quality of FASTQ files (trim_galore.py -i ./rawdata/sample.fq.gz). Then Star (v-2.6.1b) (Dobin et al. 2013) was used to align the trimmed reads to the mm9 reference (star2.pl ./index/STAR/ ./Reads/sample.fq.gz -e -p 16 -g ./Genes.gtf). Gene-specific read counts were obtained using Htseq-count (version 2.0.1) (Srinivasan et al. 2020) (htseq-count.pl -o Counts -r ./Genes.gtf ./mapping/Bams/*.bam -l). Differentially expressed genes were detected with R package EdgeR (version 3.16.5) with default parameters (Robinson et al. 2010).

### Definition of enhancers

According to a previous study (Li et al. 2023), the identification of SEs was performed based on H3K27ac ChIP-seq data using the rank ordering of super-enhancers (ROSE) algorithm (https://github.com/stjude/ROSE). Subsequently, additional filtering was applied based on specific criteria: the remaining H3K27ac peaks were classified as putative enhancers. Enhancers within 12.5 kb of each other were then stitched together, scored, and ranked according to their H3K27ac ChIP-seq signals. The resulting enhancers were plotted with enhancer rank versus enhancer density, and SEs were determined as all regions above the inflection point of the curve. A total of 1699 SEs were identified, and 66,111 typical enhancers were identified.

RYBP-targeted SE refers to a super-enhancer region that has at least one RYBP peak deposited on it.

### Protein-protein interaction analysis

The protein interactome of RYBP was analyzed using BioGRID (https://thebiogrid.org/) (Oughtred et al. 2021).

### Disease analysis

The relationship between RYBP and disease was analyzed at the website(https://www.disgenet.org/search) and GEPIA (Tang et al. 2017).

### Statistics and reproducibility

All statistical analyses were performed using R script (version 3.6.1). For histograms, two-tailed or one-tailed Welch’s t-test were used to calculate the p-value between two groups. For boxplots, one-tailed Wilcoxon test was employed. For the curve graphs, Kolmogorov-Smirnov (K-S) test were utilized. Detailed replicates and testing methods for each figure were described in the figure legends. Results were considered statistically significant when p-values were less than 0.05. Significance levels were denoted as follows: ns, not significant (*P* > 0.05); *P < 0.05; **P < 0.01; ***P < 0.001.

## Results

### RYBP occupies super-enhancers in embryonic stem cells

**Fig. 1:**
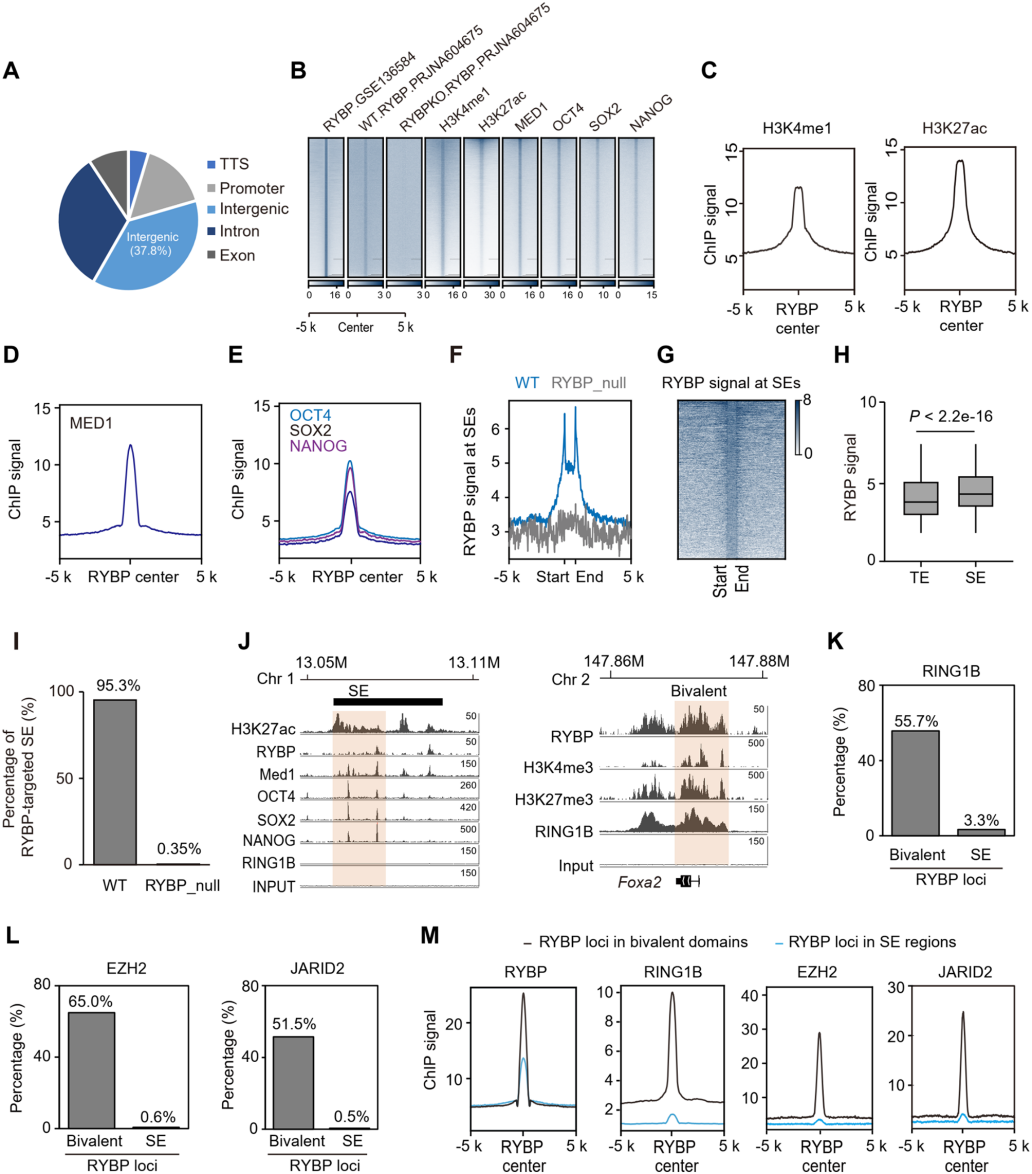
RYBP occupies super-enhancers in embryonic stem cells. (**A**) Distribution of RYBP binding sites at genomic regions. (**B**) Heatmaps showing RYBP binding loci are enriched by the histone modifications and transcription factors associated with active enhancers. (**C-E**) ChIP-seq signal of H3K4me1, H3K27ac, MED1, OCT4, SOX2 and NANOG near the RYBP peak center. (**F-G**) ChIP-seq signal of RYBP at SEs. (**H**) Boxplot showing the RYBP signal at TE and SE, one-tailed Wilcoxon test. (**I**) Histogram showing percentage of the super-enhancer regions with RYBP binding in ESCs. (**J**) Representative loci showing the ChIP-seq signal at SE and bivalent regions. (**K**) Percentage of RING1B peaks at RYBP loci within bivalent and SE regions. (**L**) Percentage of EZH2 and JARID2 peak at RYBP loci within bivalent and SE regions. (**M**) ChIP-seq signal of RYBP, RING1B, EZH2 and JARID2 at bivalent and SE regions

Several components of RYBP-defined non-canonical PRC1 exhibit transcriptional activation function, including PCGF6 and WDR5 (Ang et al. 2011; Gao et al. 2012; Huang et al. 2019). Our previous studies have demonstrated that RYBP is enriched at transcriptionally active sites, facilitating long-range interactions between active genes (Wei et al. 2022). To investigate the mechanism of RYBP-associated transcription activation, the distribution of RYBP across the entire genome in ESCs was first analyzed. As a transcription repressor (Tavares et al. 2012), 15.8% of RYBP deposits at promoter regions, and a total of 41.7% of RYBP localizes within gene bodies, including exons and introns (Fig. 1A). Remarkably, a substantial portion (37.8%) occupies intergenic regions (Fig. 1A), indicating the potential enhancer function of RYBP. The enrichment of active enhancer histone modifications, H3K4me1 and H3K27ac (Kubo et al. 2024), at RYBP-deposited loci across chromatin was observed (Fig. 1B-C). To determine the specificity of RYBP at transcriptional activation sites, the same RYBP antibody used to generate ChIP-seq data was employed for immunofluorescence in RYBP-depleted ESCs. In WT ESCs, robust RYBP signal was observed in the nucleus, whereas minimal RYBP signal was detected in RYBP-depleted ESCs (Fig. S1A-B). An independent set of RYBP ChIP-seq data was then utilized to confirm RYBP’s specific binding to chromatin. At loci where RYBP was present in WT ESCs, minimal RYBP signals were detected in RYBP knockout cells (Fig. 1B). At co-binding loci of RYBP and H3K4me1, minimal RYBP signal was observed in RYBP-deficient ESCs (Fig. S1C). Similar results were found at co-binding loci of RYBP and H3K27ac (Fig. S1D). Furthermore, four sets of RYBP ChIP-seq data, generated using three different commercial RYBP antibodies, were analyzed to confirm the deposition of RYBP at H3K4me1 or H3K27ac-binding loci (Fig. S1E-F). Therefore, RYBP deposits at H3K27ac or H3K4me1-binding loci.

Super-enhancer is a cluster of neighboring enhancers with high enhancer activity that regulates the expression of cell-type specific genes, and are marked by high density of H3K27ac, MED1 and master regulators, such as OCT4, SOX2 and NANOG in ESCs (Whyte et al. 2013). All these markers were observed to be localized at RYBP peaks (Fig. 1B-E), along with RYBP deposition at SEs (Fig. 1F-G). Compared to typical enhancer (TE), RYBP exhibits statistically significantly higher signal on SEs than that at TEs, but the difference is not substantial (Fig. 1H). In the whole genome, a total of 95.3% SEs in ESCs were occupied by RYBP (Fig. 1I). After RYBP depletion, only 0.35% of SEs were targeted by RYBP, which may be attributed to random peaks in RYBP-deficient cells (Fig. 1F and I). For instance, a SE at chromosome 1 exhibited RYBP deposition along with master regulators (Fig. 1J).

Given RYBP’s classical role as a PcG component, we wondered whether RYBP cooperates with other PcG components to bind at SEs. In ESCs, PRC1 colocalizes with PRC2 on the genome, forming bivalent domains that silence lineage genes (Ku et al. 2008). RING1B is the core catalytic subunit of PRC1 (Rose et al. 2016). Within bivalent domains, 55.7% of RYBP-deposited sites were occupied by RING1B, whereas this co-occupancy was minimal at SE regions (Fig. 1K). For instance, RING1B is enriched around the transcription start site within a bivalent domain containing the *Foxa2* gene, while it is rarely enriched at the SE (Fig. 1J). Similarly, EZH2, a core component of PRC2 (Meng et al. 2020), 65.0% of the RYBP-deposited sites at bivalent domains were occupied by EZH2, while this percentage at SE regions was only 0.6% (Fig. 1L). The similar result was also observed for another PRC2 component, JARID2 (Fig. 1L). Globally, the ChIP-seq signal of RYBP, RING1B, EZH2 and JARID2 at RYBP-deposited sites within SEs was lower than that at bivalent domains (Fig. 1M). To further confirm the enrichment of RYBP on SEs, ChIP-seq data generated using different commercial RYBP antibodies were re-analyzed. The results showed enriched RYBP signals at SEs and bivalent regions (Fig. S1G-S1H). PRC1 subunits, including CBX7, RING1B, CBX2 and PHC1, were predominantly deposited at RYBP-bound loci within bivalent regions, while minimal signals for these subunits were observed at RYBP-bound loci within SEs (Fig. S1G-H). In summary, RYBP localizes to SE regions.

### RYBP depletion impairs the enrichment of H3K27ac at SEs

**Fig. 2:**
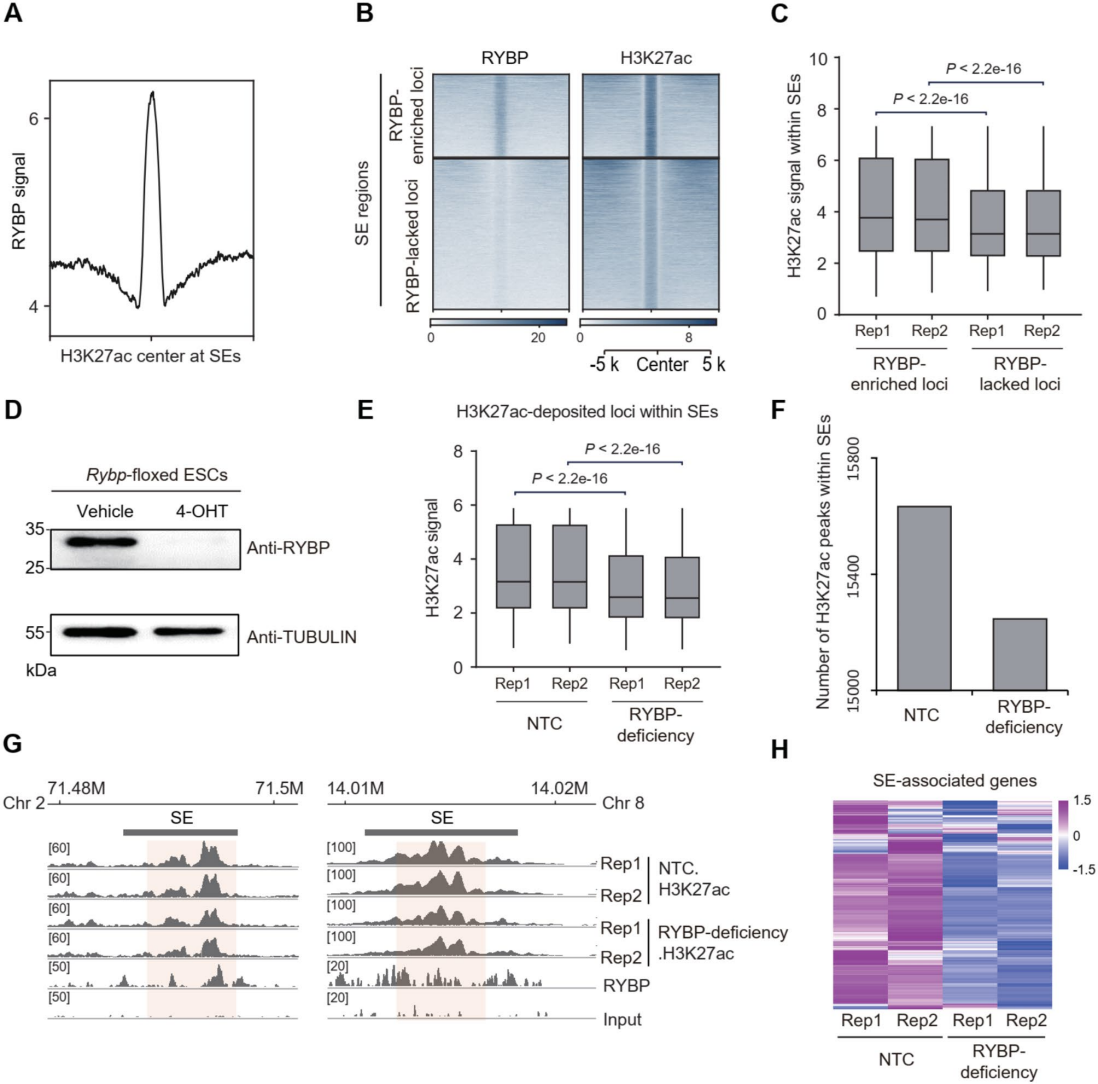
RYBP depletion impairs the enrichment of H3K27ac at SEs. (**A**) ChIP-seq signal of RYBP at H3K27ac center within SEs. (**B-C**) Heatmap and boxplot showing the H3K27ac signal at RYBP-enriched and RYBP-lacked loci within SEs, one-tailed Wilcoxon test, 2 replicates for the two groups. RYBP-enriched loci refer to the regions within SEs where RYBP and H3K27ac co-localize. RYBP-lacked loci are those regions that are marked by H3K27ac but lack RYBP deposition in SEs. (**D**) Western blot showing the expression of RYBP in *Rybp*-floxed ESCs without (vehicle) and with 4-OHT treatment. (**E**) Boxplot showing the H3K27ac signal within SEs before and after RYBP deficiency, one-tailed Wilcoxon test, 2 replicates for the two groups. (**F**) The number of H3K27ac peaks at SEs before and after RYBP depletion. (**G**) Representative loci showing the H3K27ac signal before and after RYBP deficiency. (**H**) Expression of SE-associated genes before and after RYBP deficiency

To investigate whether RYBP regulates SE activity in ESCs, the role of RYBP in H3K27ac deposition was evaluated, because enhancer activity requires H3K27ac (Raisner et al. 2018). ChIP-seq analysis revealed the enrichment of RYBP at H3K27ac-marked loci within SEs (Fig. 2A). Compared to the RYBP-lacked loci within SEs, H3K27ac signal at RYBP-enriched loci was significantly higher (Fig. 2B-C). Therefore, RYBP-deposited loci within SEs tend to be highly enriched with H3K27ac.

Subsequently, the genomic deposition of H3K27ac following RYBP deficiency was evaluated. RYBP depletion was performed via addition of 4-hydroxytamoxifen (4-OHT) in mouse *Rybp*-floxed ESCs (Fig. 2D). In the whole genome, RYBP deficiency globally reduced the H3K27ac signal at both RYBP-bound and non-RYBP-bound regions (Fig. S2A-B). Within SE regions, RYBP deficiency led to a significant reduction in H3K27ac deposition levels (Fig. 2E), as well as the number of H3K27ac peaks (Fig. 2F). A similar result was also observed at TEs (Fig. S2C). However, the reduction in H3K27ac signal at SEs after RYBP depletion is significantly greater than the reduction observed at TEs (Fig. S2D). For instance, RYBP co-localized with H3K27ac at two SEs at chromosome 2 and 8, respectively (Fig. 2G). The depletion of RYBP reduced its enrichment at these loci (Fig. 2G). In addition, the expression of numerous SE-associated genes also decreased (Fig. 2H). In summary, RYBP depletion impairs the enrichment of H3K27ac at SEs.

### RYBP depletion reduces the expression of seRNAs

**Fig. 3:**
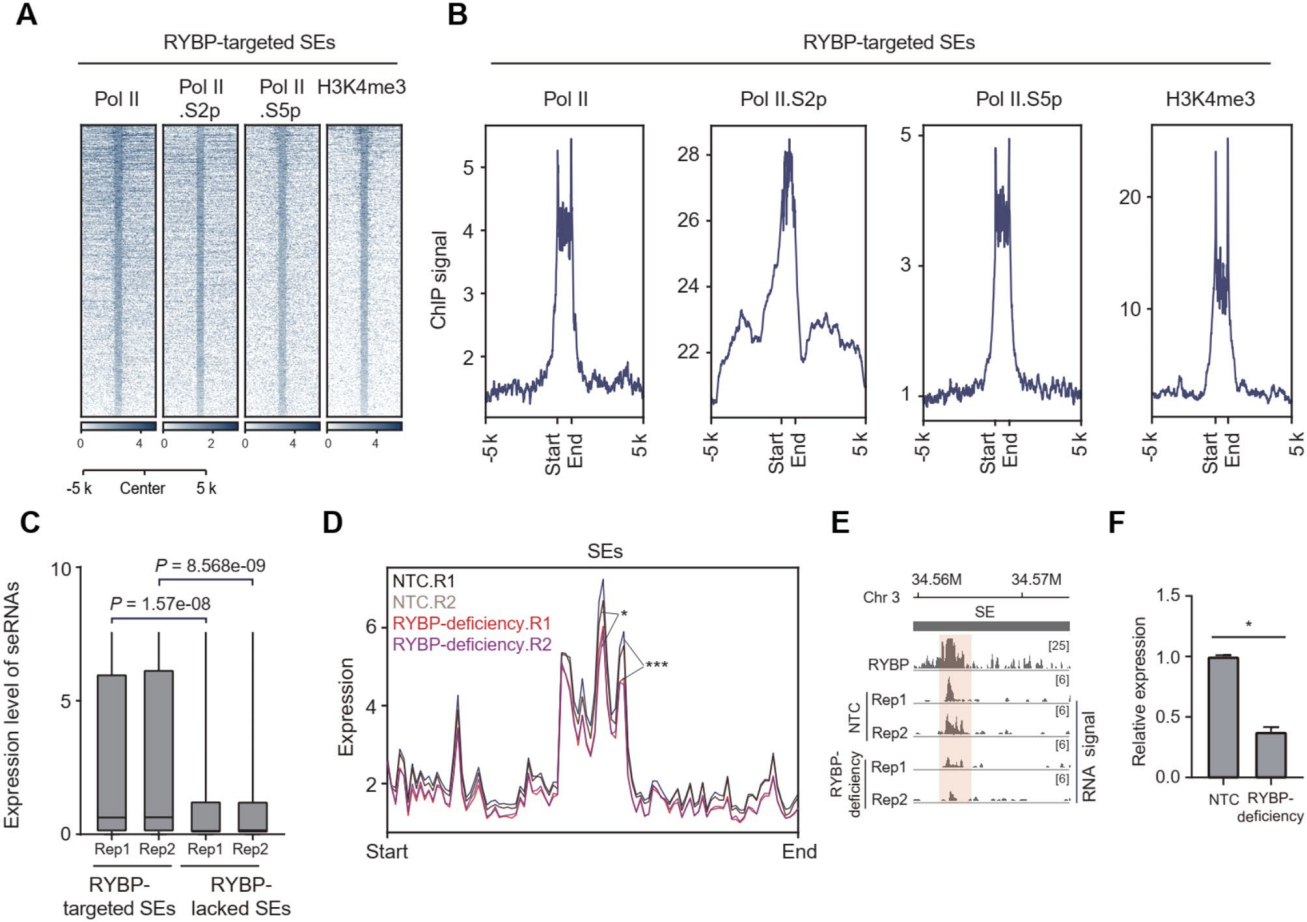
RYBP depletion reduces the expression of seRNAs. (**A**) Heatmap showing the ChIP-seq signals of Pol II (GSM515667), Pol II.S2p (GSM515663), Pol II.S5p and H3K4me3 at RYBP-targeted SEs. (**B**) The ChIP-seq signals of Pol II (GSM515667), Pol II.S2p (GSE161993), Pol II.S5p and H3K4me3 at RYBP-targeted SEs. (**C**) Boxplot showing the RNA expression levels at SEs with or without RYBP binding, one-tailed Wilcoxon test, 2 replicates for the two groups. (**D**) Quantification showing the RNA expression levels at SEs before and after RYBP deficiency, two-tailed K-S test, 2 replicates for the two groups. (**E-F**) Representative locus showing the RNA expression level at SEs before and after RYBP deficiency, one-tailed Welch’s t-test, 2 replicates for the two groups

Enhancer RNA (eRNA) transcribed from super-enhancers are able to regulates the expression of distal genes (Ding et al. 2015), we next investigated whether RYBP deficiency affects the transcription of enhancer RNA at SE regions (seRNA). RNA Pol II contributes to the generation of eRNA (Austenaa et al. 2015). Meanwhile, phosphorylated Ser 5 residues (Pol II.S5p) and Pol II with phosphorylated Ser 2 residues (Pol II.S2p) and are responsible for transcription initiation and elongation, respectively (Abe et al. 2022; Austenaa et al. 2015; Ng et al. 2003). The deposition of Pol II, Pol II.S5p and Pol II.S2p at RYBP-targeted SEs was observed (Fig. 3A-B), especially the deposition of Pol II.S2p at RYBP-targeted SEs indicates the RNA elongation at enhancers (Fig. 3A-B). Results from multiple sets of Pol II and Pol II.S2p ChIP-seq data revealed extended signals beyond SE regions (Fig. S3A-B), possibly due to their elongation function across DNA (Austenaa et al. 2015). Moreover, transcribed enhancers typically exhibit high levels of H3K4me3 deposition (Djebali et al. 2012), and the enrichment of H3K4me3 at RYBP-targeted SEs were also observed (Fig. 3B). These results indicate the transcriptional events at RYBP-targeted SEs. Compared to the SEs lacking RYBP, RYBP-targeted SEs exhibited a higher level of RNA expression (Fig. 3C). Following RYBP deficiency, the expression of RNA at TEs did not significantly reduce (Fig. S3C), but their expression at SEs significantly decreased (Fig. 3D-F, S3D-E). Altogether, RYBP depletion reduces seRNA expression.

### RYBP co-localizes with TrxG components at SEs

**Fig. 4:**
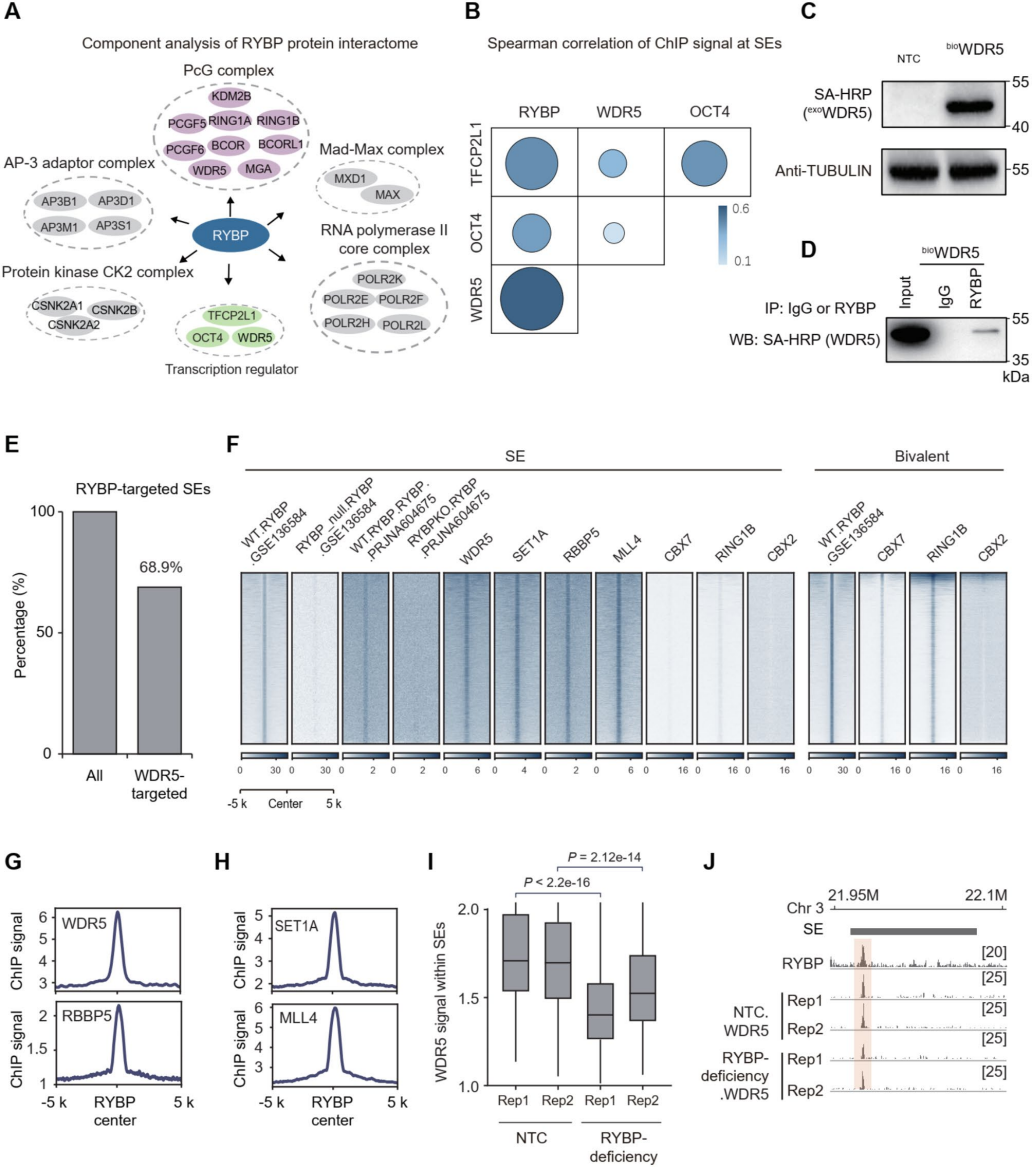
RYBP co-localizes with TrxG components at SEs. (**A**) Cell component analysis of RYBP protein interactome. (**B**) Spearman correlation between different ChIP signals at SEs. (**C**) Western blot showing the expression of biotin-tagged WDR5 (^bio^WDR5) in ESCs. (**D**) Co-IP showing the interaction between RYBP and WDR5. (**E**) Histograms showing percentage of WDR5-targeted SEs among all the RYBP-targeted SEs in mESCs. (**F-H**) ChIP-seq signal of TrxG and PcG components at the RYBP peak center. (**I**) Boxplot showing the WDR5 signal at SEs before and after RYBP deficiency, one-tailed Wilcoxon test, 2 replicates for the two groups. (**J**) Representative locus showing the WDR5 signal at SE before and after RYBP deficiency

To investigate the mechanism of RYBP in regulating SE activity, the protein interactome of RYBP was first investigated. As a component of PcG, many proteins of PcG complex within RYBP interactome were observed (Fig. 4A). Proteins with transcriptionally active function were also observed, including transcription factors TFCP2L1 and OCT4 (Fig. 4A). In addition, WDR5, the core component of TrxG which catalyzes H3K4me3 for transcription activation (van Nuland et al. 2013), was also observed (Fig. 4A). Compared to TFCP2L1 and OCT4, WDR5 exhibits the highest correlation with RYBP in terms of enriched signals on SEs (Fig. 4B). Then co-IP was further performed in biotin-tagged WDR5 ESCs (Fig. 4C), which also demonstrated the physical interaction between RYBP and WDR5 (Fig. 4D).

Subsequently, whether RYBP co-occupied with TrxG components at SEs were investigated, and the deposition of WDR5 within SEs was firstly detected. WDR5 was found to be deposited at typical enhancer regions in ESCs (Fig. S4A-B), and co-localized with SE-associated histone modifications and marker proteins, including H3K27ac, MED1, OCT4, SOX2 and NANOG (Fig. S4C-D). Moreover, WDR5 enrichment at RYBP-targeted SEs was also observed (Fig. S4E). In total, 68.9% of RYBP-targeted SEs were occupied by WDR5 (Fig. 4E). Within SEs, the deposition of WDR5 at RYBP loci was observed (Fig. 4F). Other TrxG components including RBBP5, SET1A and MLL4, are also enriched at RYBP-targeted SEs (Fig. S4E), as well as at RYBP-deposited loci within SEs (Fig. 4F-H). The minimal RYBP signal at SEs in RYBP-depleted cells confirmed the specific binding of RYBP at SEs in WT ESCs (Fig. 4F). Compared to bivalent regions, minimal deposition of PRC1 proteins, including CBX7, RING1B and CBX2, was observed at RYBP-targeted loci within SEs (Fig. 4F, S4F). Thus, RYBP co-localized with TrxG components at SEs.

The impact of RYBP-deficiency on WDR5 occupation at SEs was further analyzed. The intensity of WDR5 signal at numerous loci within SEs decreased after RYBP depletion (Fig. 4I). For instance, at a SE region within chromosome 3 where RYBP co-localized with WDR5, RYBP-deficiency resulted in decreased WDR5 deposition in this region (Fig. 4J). Altogether, RYBP co-occupies with TrxG component WDR5 at SEs, RYBP-deficiency reduced WDR5 deposition at SEs.

### Deficiency of TrxG component reduces the expression of RYBP-regulated seRNAs

**Fig. 5:**
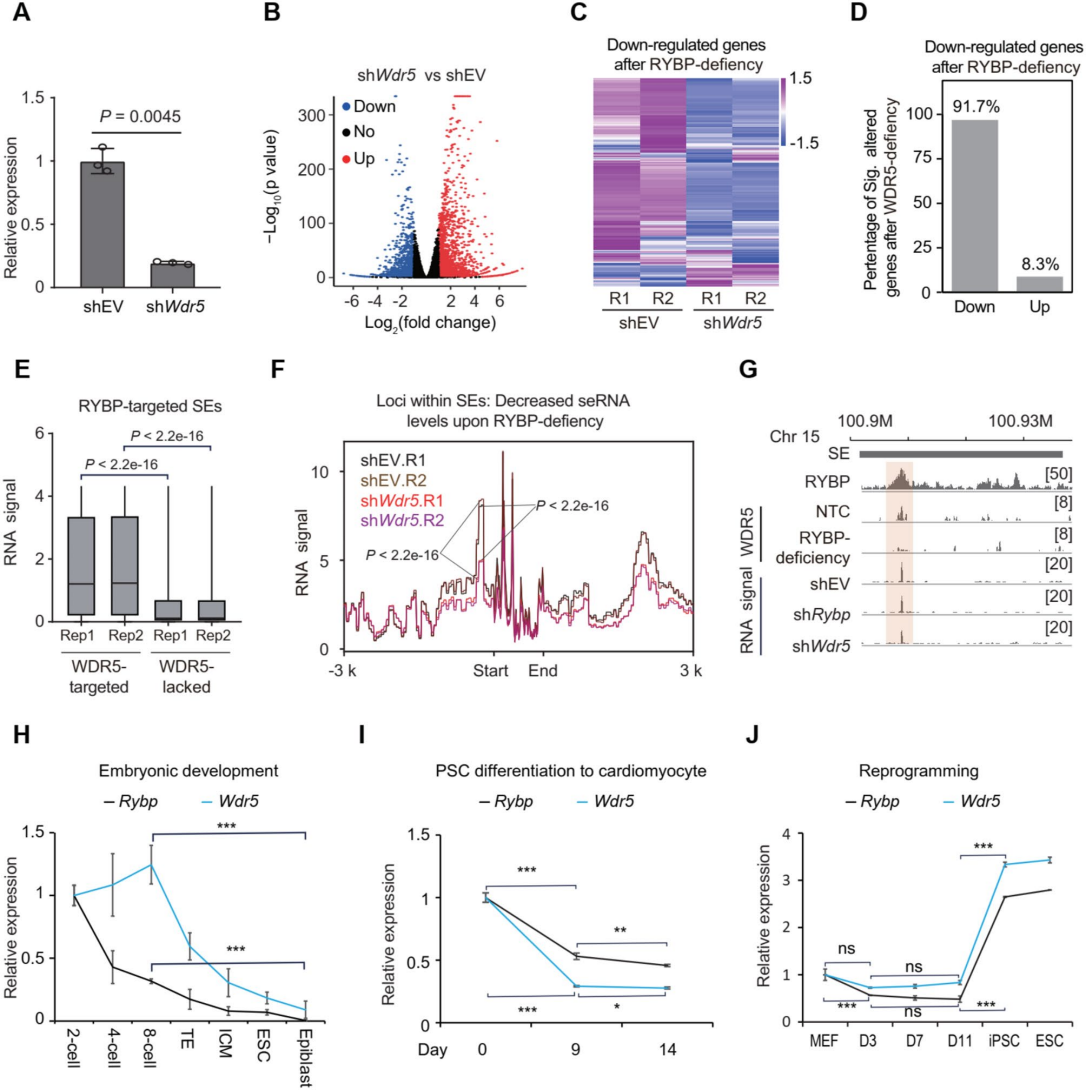
Deficiency of TrxG component reduces the expression of RYBP-regulated seRNAs. (**A**) Relative expression of *Wdr5* after WDR5 knockdown, two-tailed Welch’s t-test, *n* = 3. (**B**) Volcano plot showing the expression alteration of genes before and after WDR5 knockdown, 2 replicates for the two groups. (**C**) The expression alteration of genes before and after WDR5 knockdown, the gene set was the significantly down-regulated genes upon RYBP deficiency, 2 replicates for the two groups. (**D**) The percentage of down and up-regulated genes upon WDR5 knockdown, the gene set was the significantly down-regulated genes upon RYBP-deficiency, and also significantly changed after WDR5 knockdown. (**E**) Boxplot showing the RNA expression levels at RYBP-targeted SEs with or without WDR5 binding, one-tailed Wilcoxon test, 2 replicates for the two groups. (**F**) The RNA expression levels at SEs after WDR5 knockdown, the loci in SEs with decreased RNA expression after RYBP-deficiency were detected, two-tailed K-S test, 2 replicates for the two groups. (**G**) The representative locus showing the WDR5 deposition at chromatin and RNA expression levels at SEs in different groups. (**H**) The expression tendency of *Rybp* and *Wdr5* mRNA during embryonic development. Two-tailed Welch’s t-test, the replicates in each point from 2-cell to epiblast were 8, 6, 6, 2, 9, 13 and 3, respectively, the data were re-analyzed from public study (PMID: 21731673). (**I**) The expression tendency of *Rybp* and *Wdr5* mRNAs during PSC differentiation to cardiomyocyte. Two-tailed Welch’s t-test, and 4 replicates for each point. (**J**) The expression tendency of *Rybp* and *Wdr5* mRNAs during reprogramming. Two-tailed Welch’s t-test, the replicates in each point from MEF to iPSC were 2, 3, 3, 3, 2 and 2, respectively

We next investigated whether RYBP coordinates with WDR5 to facilitate seRNA expression, and initially explored their synergistic regulatory effects on general gene transcription. The knockdown of WDR5 was performed via shRNA (Fig. 5A, S5A), and subsequent changes in the expression of numerous genes (Fig. 5B). Regarding genes down-regulated upon RYBP-deficiency, WDR5 knockdown also led to significant expression alterations of these genes, with 91.7% being down-regulated and only 8.3% up-regulated (Fig. 5C-D). These results suggested that RYBP might coordinate with WDR5 to regulate gene transcription.

Subsequently, the synergistic regulatory effects of RYBP and WDR5 on seRNA expression were further investigated. Among RYBP-targeted SEs, the expression level of seRNA at WDR5-targeted SEs was significantly higher than that of WDR5-lacked SEs (Fig. 5E). Regarding loci with decreased seRNA expression upon RYBP deficiency, their expression also tended to be down-regulated (Fig. 5F). As an example, RYBP co-localized with WDR5 within a SE at chromosome 15, RYBP deficiency reduced WDR5 deposition at this site, as well as seRNA expression, while WDR5 knockdown also reduced seRNA expression at this site (Fig. 5G). Therefore, WDR5 is required for the expression of RYBP-regulated seRNA.

Because SEs usually control the expression of cell-fate associated genes (Whyte et al. 2013), the expression alteration patterns of RYBP and WDR5 during cell fate transitions were analyzed. During multiple cell fate transitions, similar expression trends for *Rybp* and *Wdr5* mRNAs were observed (Fig. 5H-J). During embryonic development, the expression of both *Rybp* and *Wdr5* mRNAs significantly down-regulated from the 8-cell stage to the epiblast stage (Fig. 5H). During the differentiation of pluripotent stem cells (PSCs) into cardiomyocytes, the expression of both *Rybp* and *Wdr5* mRNAs also significantly down-regulated (Fig. 5I). During the reprogramming of mouse embryonic fibroblasts (MEFs) into induced pluripotent stem cells (iPSCs), the expression of *Rybp* and *Wdr5* mRNAs did not significantly change from Day 3 to Day 11, but significantly up-regulated when they transformed into iPSCs (Fig. 5J). Western blotting results confirmed the decreased expression of RYBP and WDR5 proteins during the differentiation of ESCs into cardiomyocytes (Fig. S5B) and the increased expression of them during somatic cell reprogramming (Fig. S5C). In summary, deficiency of the TrxG component reduced the expression of RYBP-regulated seRNA, suggesting that RYBP and WDR5 synergistically activate seRNA expression.

### RYBP depletion impairs TrxG-associated H3K4me3 at SEs

**Fig. 6:**
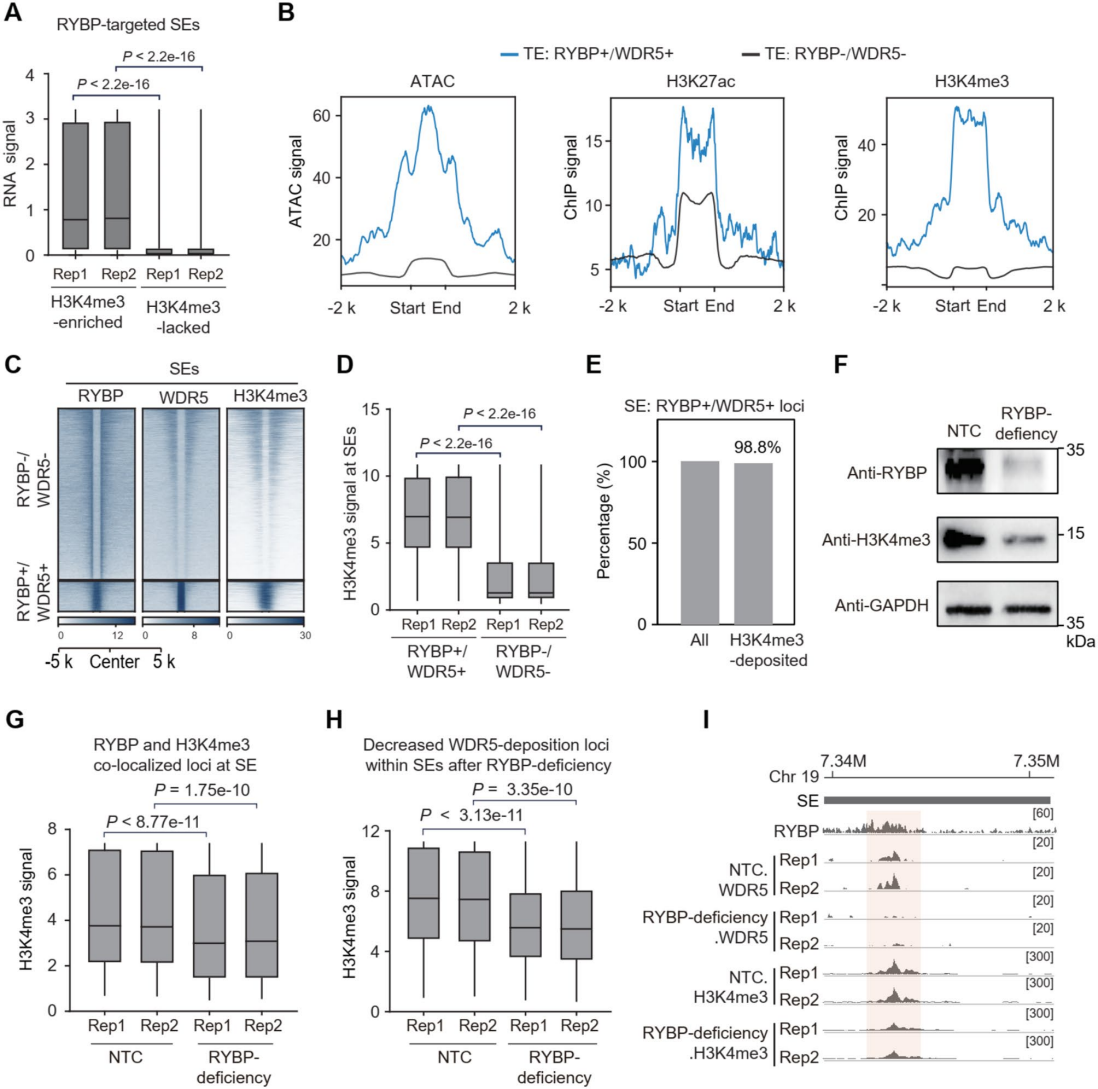
RYBP depletion impairs TrxG-associated H3K4me3 at SEs. (**A**) Boxplot showing the RNA expression levels at RYBP-targeted SEs with or without H3K4me3 deposition, Wilcoxon test. (**B**) ATAC, H3K27ac and H3K4me3 signals at typical enhancers with (RYBP+/WDR5+) or without (RYBP-/WDR5-) both RYBP and WDR5 deposition. (**C-D**) Heatmap and boxplot showing the H3K4me3 signals at both RYBP and WDR5-lacked loci (RYBP-/WDR5-), as well as at the both RYBP and WDR5-deposited loci (RYBP+/WDR5+), one-tailed Wilcoxon test, 2 replicates for the two groups. (**E**) Among the RYBP and WDR5 co-localized loci within SE, the percentage of these loci with H3K4me3 deposition was displayed. (**F**) Western blot showing the levels of H3K4me3 in ESCs after RYBP depletion. (**G**) Boxplot showing the H3K4me3 signals at RYBP and H3K4me3 co-localized regions within SEs before and after RYBP deficiency, one-tailed Wilcoxon test, 2 replicates for the two groups. (**H**) Boxplot showing the H3K4me3 signal at loci within SEs, the deposition of WDR5 at these loci decreased after RYBP-deficiency, one-tailed Wilcoxon test, 2 replicates for the two groups. (**I**) The representative locus showing the WDR5 and H3K4me3 deposition at SE before and after RYBP deficiency

TrxG primarily catalyzes H3K4me3 (van Nuland et al. 2013), which positively correlates with eRNA expression (Djebali et al. 2012). A similar result was also revealed for the expression of seRNAs at RYBP-targeted SEs, where the regions with H3K4me3 deposition showed significantly higher levels of seRNA expression than those lacking H3K4me3 (Fig. 6A). Because RYBP interacts with TrxG component WDR5 (Fig. 4D), which is a scaffold of TrxG and required for the deposition of H3K4me3 (Ang et al. 2011; Guarnaccia and Tansey 2018). Therefore, we wondered whether RYBP coordinates with WDR5 for H3K4me3 deposition at SEs. In comparison to the TEs without RYBP and WDR5, TEs with both RYBP and WDR5 deposition showed higher ATAC signals (Fig. 6B), indicating an open chromatin state for transcription activation. Regarding the histone modifications of TEs, TEs with both RYBP and WDR5 displayed higher signals of H3K27ac and H3K4me3 compared to those lacking RYBP and WDR5 (Fig. 6B). In SE regions, there is a significantly higher H3K4me3 intensity at sites where both RYBP and WDR5 co-localized than that at the site simultaneous absence of RYBP and WDR5 (Fig. 6C-D). Regarding RYBP and WDR5 co-localized loci within SE regions, nearly all (98.8%) these loci were enriched with H3K4me3 peaks (Fig. 6E). Therefore, H3K4me3 tends to be highly enriched at RYBP and WDR5 co-localized loci at SEs.

Whether RYBP deficiency affects H3K4me3 deposition at SE regions was next investigated. RYBP depletion reduced the global accumulation of H3K4me3 in ESCs (Fig. 6F), as well as its deposition at numerous RYBP and H3K4me3 co-localized loci within SEs (Fig. 6G). Furthermore, numerous sites with decreased WDR5 deposition upon RYBP deficiency, also showed a tendency for decreased H3K4me3 enrichment (Fig. 6H). As an example, RYBP co-localized with WDR5 and H3K4me3 at a SE region within chromosome 19, RYBP deficiency led to reduced deposition of both WDR5 and H3K4me3 (Fig. 6I). Therefore, RYBP depletion impairs the enrichment of H3K4me3 at SEs.

### RYBP is involved in intra and inter SE interactions

**Fig. 7:**
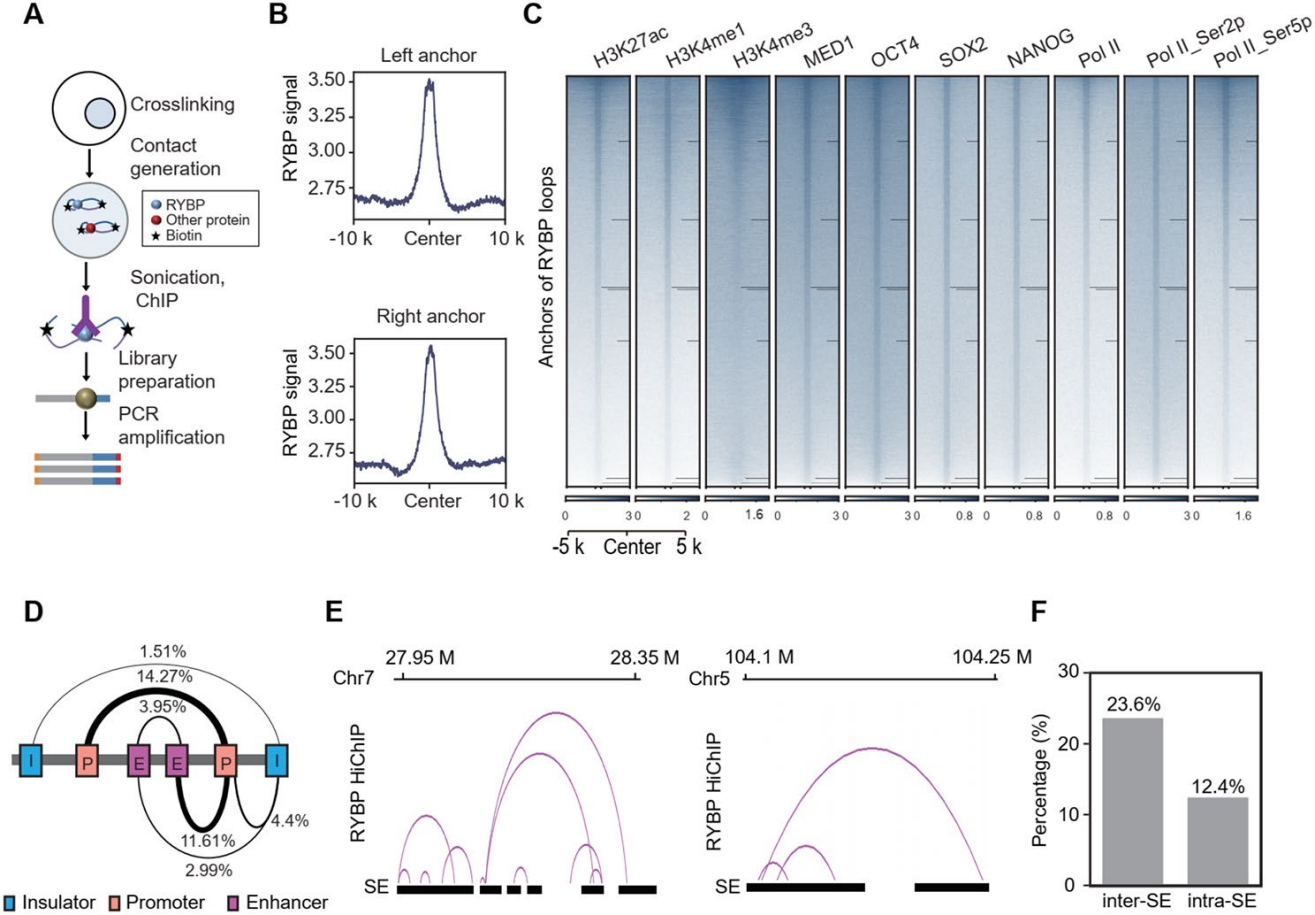
RYBP is involved in intra and inter SE interactions. (**A**) Experimental pipeline for RYBP HiChIP. (**B**) RYBP ChIP-seq signal at anchors of RYBP HiChIP data. (**C**) Heatmaps showing the signals of different proteins at the anchors of RYBP HiChIP data. (**D**) The percentage of interactions between different DNA elements from RYBP HiChIP data. (**E-F**) The representative loci and quantification showing the RYBP-associated interactions intra or inter SEs

Reported work has revealed that multiple enhancers are nested via 3D chromatin interactions for transcription control (Lin et al. 2022), previous study also revealed that RYBP contributes to long-range chromatin interactions (Wei et al. 2022), we therefore wondered whether RYBP is involved in the interactions between SEs. Using RYBP HiChIP, we identified RYBP-associated interactions (Fig. 7A). The deposition of RYBP ChIP signal at the interacted anchors confirms the validity of the HiChIP data (Fig. 7B).

Globally, the histone modifications and transcription factors associated with SE are enriched at the anchors of RYBP-associated interactions, as well as the total and phosphorylated RNA pol II (Fig. 7C). RYBP participates in the interactions between various DNA elements, including insulator, enhancer and promoter, with RYBP involved in 3.95% of enhancer-enhancer interactions (Fig. 7D). Regarding SEs, RYBP was found to participate in interactions both within and between SEs (Fig. 7E). Overall, 23.6% and 12.4% of SEs engage in inter- and intra-SE interactions, respectively (Fig. 7F).

### RYBP generally occupies SEs across cell types and species

**Fig. 8:**
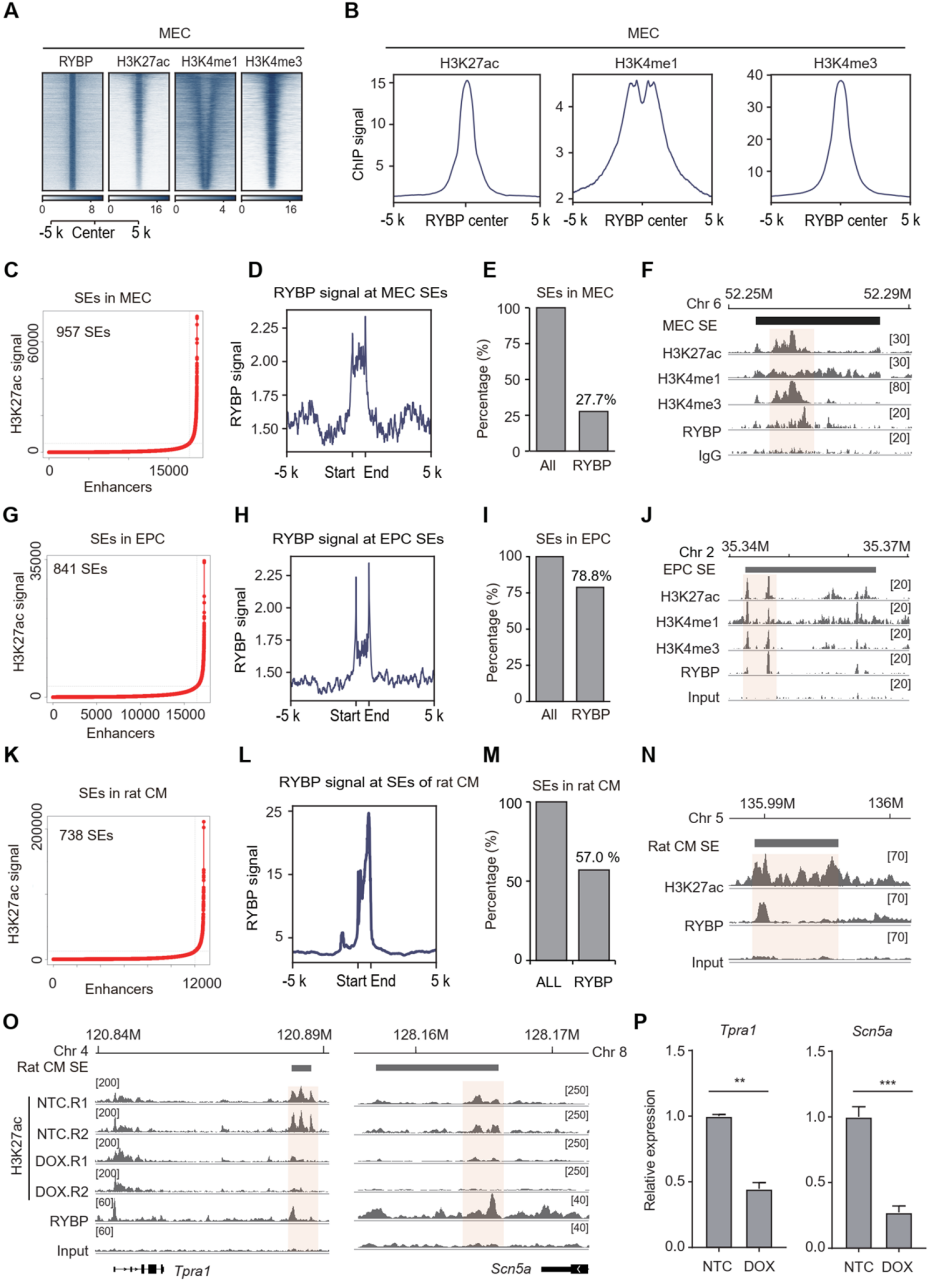
RYBP generally occupies SEs across cell types and species. (**A-B**) H3K27ac, H3K4me1 and H3K4me3 signals at RYBP loci in MECs. (**C**) H3K27ac signals at enhancers in MECs. (**D**) RYBP signals at SEs in MECs. (**E**) The percentage of RYBP-targeted SEs among all SEs in MECs. (**F**) The representative loci showing the deposition of RYBP and histone modification at SE in MECs. (**G**) H3K27ac signal at enhancers in EPCs. (**H**) RYBP signal at SEs in EPCs. (**I**) The percentage of RYBP-targeted SEs among all SEs in EPCs. (**J**) The representative loci showing the deposition of RYBP and histone modifications at SE in EPCs. (**K**) H3K27ac signals at enhancers in rat CM. (**L**) RYBP signals at SEs in rat CM. (**M**) The percentage of RYBP-targeted SEs among all SEs in rat CM. (**N**) The representative loci showing the deposition of RYBP and histone modification at SE in rat CM. (**O**) The representative loci showing the H3K27ac signals before or after DOX treatment in rat CM. (**P**) The expression alteration of *Tpra1* and *Scn5a* after DOX treatment

We then wondered whether RYBP generally occupies SEs across different types of cells and species. ChIP-seq data from another in vitro differentiated cells, mesodermal cells (MECs) were analyzed. Histone modifications associated with active enhancers, including H3K27ac, H3K4me1 and H3K4me3, deposited at RYBP binding loci in MECs (Fig. 8A-B). A total of 957 SEs were identified by H3K27ac signal in MECs (Fig. 8C), and RYBP was found to be enriched at SEs in MECs (Fig. 8D). Specifically, 27.7% of SEs in MECs were targeted by RYBP (Fig. 8E). For example, in MECs, RYBP signals were observed at a SE on chromosome 6 (Fig. 8F). Thus, the occupation of RYBP at SEs is not limited to ESCs but extends to differentiated cells.

To investigate whether RYBP occupies SEs in cells derived from in vivo tissues, ChIP-seq data for RYBP and histone modifications were analyzed from newborn mice-derived epidermal progenitor cell (EPC) (Cohen et al. 2018). H3K27ac, H3K4me1 and H3K4me3 deposited at RYBP binding loci in both EPCs (Fig. S6A-S6B). A total of 841 SEs were identified by H3K27ac signal in EPCs (Fig. 8G), and 78.8% of SEs in EPCs were targeted by RYBP (Fig. 8H-I). As an example, in EPCs, a SE on chromosome 2 exhibited deposition of H3K27ac, H3K4me1, and H3K4me3, with concurrent enrichment of RYBP (Fig. 8J). In addition, ChIP-seq data from mouse brain-derived cells were also analyzed (Gao et al. 2014; Liu et al. 2021). A total of 287 SEs were identified (Fig. S6C), and 22% of SEs are targeted by RYBP (Fig. S6D-F). Thus, the occupation of RYBP at SEs is also observed in in vitro tissues.

To investigate whether RYBP-targeted SEs is responsible for disease process, a model of Doxorubicin (DOX)-induced heart failure was used. A total of 738 SEs were identified in cardiomyocytes (CMs) isolated from rat hearts (Fig. 8K). Notably, 57% of these SEs in rat CMs were targeted by RYBP (Fig. 8L-N). DOX treatment led to a reduction in H3K27ac signal at several RYBP-targeted SEs in rat CMs (Fig. 8O), and the expression of genes near two example SEs was significantly downregulated (Fig. 8P), suggesting impaired SE activity.

Therefore, RYBP is deposited at SEs in cells derived from both in vitro cell lines and in vivo tissue-derived cells, including both mouse and rat cells. Additionally, RYBP-targeted SEs respond to disease processes.

### RYBP is required for cell fate control

**Fig. 9:**
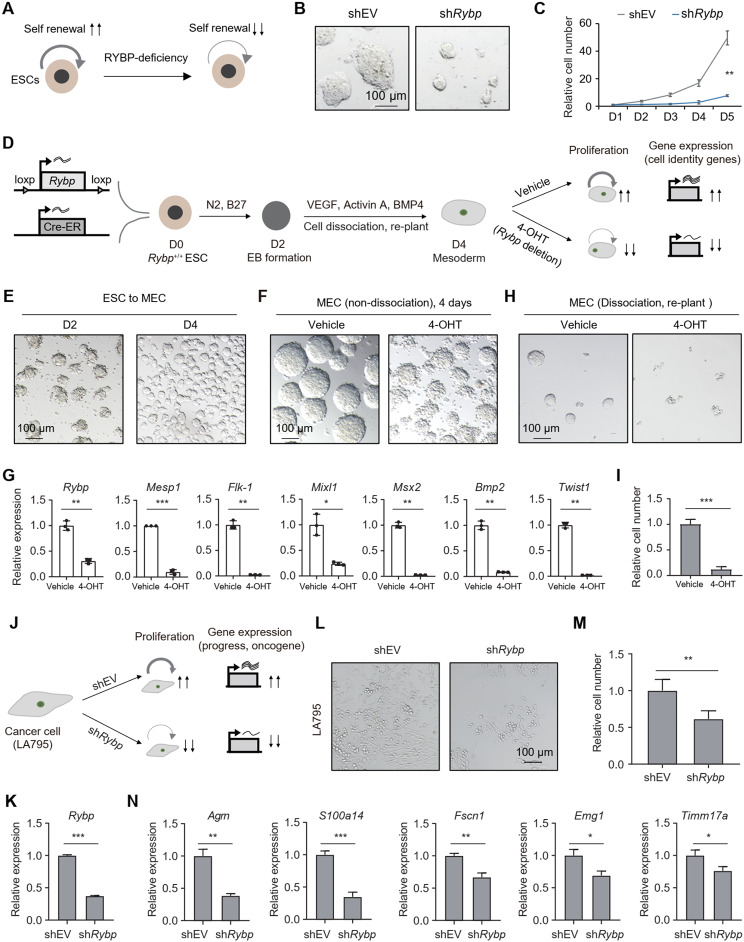
RYBP is required for cell fate control. (**A**) Schematic of the experimental design for investigating the role of RYBP in ESC maintenance. (**B**) Representative images showing the ESC colonies in different groups. (**C**) The relative number of ESCs in shEV or sh*Rybp* group across 5 days, two-tailed Welch’s t-test, 4 replicates for each group. (**D**) Schematic of the experimental design for investigating the role of RYBP in MEC maintenance. (**E**) Representative images showing the EBs at Day 2 and Day 4 during ESC differentiation. (**F**) Representative images showing the EBs in different groups, the EBs were continuously cultured without dissociation. (**G**) The expression of genes after 4 days treatment in different groups, two-tailed Welch’s t-test, 3 replicates for each group. (**H**) Representative images showing the EBs in different groups, the EBs were dissociated and re-planted. (**I**) The relative number of MECs in different groups, two-tailed Welch’s t-test, 5 replicates for each group. (**J**) Schematic of the experimental design for investigating the role of RYBP in cancer cell maintenance. (**K**) RT-qPCR detects the knockdown of *Rybp* RNA in LA795 cells. (**L**) Representative images showing the LA795 cells in different groups. (**M**) The relative number of ESCs in shEV or sh*Rybp* group after 4 days, two-tailed Welch’s t-test, 5 replicates for each group. (**N**) RT-qPCR detects the expression of genes in LA795 cells from shEV or sh*Rybp* group, two-tailed Welch’s t-test, 3 replicates for each group

Given the association of super-enhancers with cell fate regulation and their implication in human diseases (Mansour et al. 2014; Oldridge et al. 2015; Whyte et al. 2013), we first investigated whether RYBP controls cell fate during development, and experiments were conducted in ESCs and their differentiation processes. Results showed that RYBP knockdown reduced the size of ESC colonies and their proliferation ability (Fig. 9A-C), indicating that RYBP is essential for the self-renewal of ESCs. Next, the transition of ESCs towards the three germ layers was assessed using a random differentiation system via EB formation (Fig. S7A). RYBP knockout did not affect EB formation (Fig. S7B). However, the expression of genes associated with the three germ layers was significantly affected by RYBP knockout. Specifically, the ectoderm marker gene *Pax3* was significantly reduced at Day 14. At Day 4, the mesendoderm marker gene *T* was significantly reduced. The mesoderm marker gene *Mesp1* was significantly downregulated at Day 6. The endoderm marker genes *Gata4* and *Gata6* were significantly reduced on Days 14 and 10, respectively (Fig. S7C). Given that RYBP occupies SE regions in MECs (Fig. 8C-F), its role in the maintenance of MECs was further examined. RYBP-inducible depletion ESCs (Fig. 2D) were induced to differentiate into MECs in vitro for 4 days, followed by *Rybp*-deletion via 4-OHT treatment. The proliferation ability and expression of MEC-associated genes were then assessed (Fig. 9D). After 4 days of differentiation, ESCs formed MECs with normal EB morphology (Fig. 9E). Compared to vehicle group, the EB size from 4-OHT treatment group was seemingly smaller (Fig. 9F), and the expression of several MEC-associated genes, including *Mesp1*, *Flk-1*, and *Mixl1*, significantly decreased (Fig. 9G). Additionally, dissociated MECs were re-planted in dishes, and showed impaired EB re-formation ability and reduced proliferation ability upon 4-OHT treatment (Fig. 9H-I). These findings collectively suggest that RYBP is crucial for the proper development.

Then whether dysregulation of RYBP is linked to human pathologies was further investigated. Alterations in RYBP expression have been associated with various biological processes and diseases, including tumor cell invasion, Hodgkin disease, and liver carcinoma (Fig. S7D). Additionally, RYBP mutations have been implicated in changes in body height, progression of liver carcinoma, and neoplasm metastasis (Fig. S7E). Analysis of RYBP expression in tumors revealed dosage alterations in multiple tumor types (Fig. S7F). For example, RYBP is highly expressed in adrenocortical carcinoma (ACC), esophageal carcinoma (ESCA) and kidney chromophobe (KICH) (Fig. S7F), and the high level of RYBP is positively related to shorter survival rates for patients with these tumors (Fig. S7G). Conversely, RYBP is down-regulated in uterine corpus endometrial carcinoma (UCEC) and uterine carcinosarcoma (UCS) (Fig. S7F), lower RYBP levels associated with shorter survival rates for patients with these malignancies (Fig. S7H). Therefore, dysfunction of RYBP is associated with human diseases. Given that RYBP is expressed in various cancer cells (Fig. S7F), we further investigated whether RYBP is also required for the maintenance of these cells (Fig. 9J). The knockdown of *Rybp* in lung adenocarcinoma cells (LA795) was performed via shRNA (Fig. 9K), which significantly impaired their proliferation ability (Fig. 9L-M). In addition, several genes associated with the progression, migration and invasion of lung adenocarcinoma cells, significantly down-regulated upon *Rybp* knockdown (Fig. 9N). These findings indicate that RYBP is required for the maintenance of lung adenocarcinoma cells.

Altogether, RYBP is required for the cell fate control during development, as well as cancer cells. Dysregulation of RYBP is linked to human pathologies.

## Discussion

Enhancers are a class of DNA elements in the genome that can activate gene expression (Banerji et al. 1981, 1983), their regulatory effects on target genes are not limited by distance or position relative to the target gene (Banerji et al. 1981). Histone modification such as H3K4me1 is usually enriched at enhancers (Heintzman et al. 2007), and the presence of co-activators such as P300, and highly enriched histone modification H3K27ac indicates their strong transcriptional activation regulatory activity (Bonn et al. 2012; Rada-Iglesias et al. 2011). A large number of enhancers are clustered together and can span genomic distances of up to 50 kilobases, forming super-enhancers (Whyte et al. 2013). Previous research has shown that RYBP collaborates with OCT4 to activate the expression of pluripotency-associated genes, which typically govern the cell fate of stem cells (Li et al. 2017). This finding is similar to our conclusion that RYBP regulates SE activity, because SEs also control the expression of cell fate-associated genes (Whyte et al. 2013). Therefore, our study elucidates the mechanism by which Polycomb protein RYBP facilitates transcription from the perspective of super-enhancers.

Certain enhancer regions exhibit high enrichment of RNA Pol II and can transcribe eRNAs, further promoting enhancer activity and neighboring gene expression (Ding et al. 2015; Djebali et al. 2012; Hah et al. 2015; Henriques et al. 2018; Pefanis et al. 2015; Suzuki et al. 2017). eRNAs regulate the expression of distant genes through various mechanisms, such as facilitating enhancer-promoter interactions and recruiting coactivators (Jiao et al. 2018; Rahnamoun et al. 2018). Unlike promoters, eRNAs are transcribed bidirectionally from the transcription start site of enhancers (Austenaa et al. 2015). A previous study revealed that transcribed enhancers show stronger enrichment of histone modification H3K4me3 (Djebali et al. 2012), and H3K4me3 facilitates RNA transcription by promoting the elongation process of RNA Pol II (Wang et al. 2023). Thus, RYBP loss-induced reduction of H3K4me3 may be one of the reasons for the impaired eRNA transcription. In addition, the depletion of RYBP results in attenuated enrichment of H3K27ac, which may represent another reason contributing to the downregulation of eRNA expression. Transcribed enhancers display enrichment of H3K27ac (Djebali et al. 2012), and H3K27ac is required for eRNA transcription (Kang et al. 2021). Given that TrxG facilitates the deposition of both H3K27ac and H3K4me3 (Tie et al. 2009; van Nuland et al. 2013), we propose a mechanism in which RYBP collaborates with WDR5 at SEs and further promotes the deposition of various active histone modifications, thereby enhancing eRNA transcription.

The present study demonstrates that RYBP depletion reduces the chromatin deposition of WDR5 at chromatin. We did not rule out the possible indirect consequence of PRC1 dysfunction. Given that PRC1 plays a crucial role in maintaining pluripotency (Morey et al. 2012), RYBP deficiency could potentially facilitate the exit from pluripotency in ESCs (Wei et al. 2022), which might further contribute to the dissociation of WDR5 from chromatin. The reduction of H3K27ac at RYBP-lacked loci following RYBP depletion further supports this possibility. Additionally, RYBP collaborates with WDR5 at SEs, we also do not exclude the possibility that other proteins may also interact with RYBP at SEs. For instance, OCT4 has been reported to be enriched at SEs (Whyte et al. 2013). Our study revealed co-localization of RYBP and OCT4 at SE, and previous research has established a physical interaction between RYBP and OCT4 (Li et al. 2017). Therefore, it is plausible that RYBP also collaborates with OCT4 at SEs for its function.

RYBP is required for the pluripotency maintenance of embryonic stem cells (Wei et al. 2022). The classical function of RYBP is as a component of the non-canonical PRC1 to repress the expression of lineage genes. Increasing evidence suggests that RYBP also possesses transcriptional activation characteristics (Li et al. 2017; Wei et al. 2022). The present study further reveals that RYBP regulates the expression of eRNAs. The expression of transcribed enhancers and eRNAs is cell type-specific (Djebali et al. 2012), and factors determining cell fate are also involved in eRNA transcription (Pulakanti et al. 2013), suggesting a role for eRNAs in cell fate determination. In embryonic stem cells, super-enhancers can also transcribe eRNAs and regulate the expression of distal pluripotency genes, thereby maintaining stem cell pluripotency (Ding et al. 2015). In contrast to its role in inhibiting developmental gene expression dependent on Polycomb, RYBP also regulates SE activity for cell fate control, which process seemingly does not rely on Polycomb complex. Therefore, dual mechanisms by which RYBP safeguards the fate of stem cells are proposed.

Multiple mechanisms have been reported regarding the relationship between eRNA transcription and human disease. For instance, in pancreatic ductal adenocarcinoma, CFL1 overexpression enhances m6A on seRNA, recruiting MLL1 for H3K4me3 modification. This increases H3K4me3 at super-enhancers, enhancing chromatin accessibility and oncogene transcription (Li et al. 2023). In human breast cancer cells, the binding of E2 to ER-α enhances eRNA transcription at enhancers. These eRNAs appear to play a crucial role in augmenting enhancer-promoter interactions and gene activation (Li et al. 2013). Furthermore, E2 induces the recruitment of a megadalton-scale protein complex, including GATA3, which forms phase-separated liquid droplets. This phase separation facilitates long-distance interactions and cooperative activation of enhancers (Liu et al. 2014; Nair et al. 2019), and E2-induced eRNAs promote the formation of GATA3 condensates (Nair et al. 2019). These findings underscore the critical role of eRNA in phase separation-driven chromatin architecture and its implications in human disease. Studies have shown that biomolecules on super-enhancers, including transcription factors and non-coding RNAs, undergo phase separation (Hnisz et al. 2013, 2017; Sabari et al. 2018). The present reveals that RYBP regulates the expression of eRNA, and is involved in inter-SE interactions, and our previous study has indicated that RYBP can undergo phase separation and facilitates long-range interactions (Wei et al. 2022). A possible speculation is that RYBP activates the expression of eRNAs, which may facilitate the phase separation of RYBP within the regions of super-enhancers. This, in turn, could facilitate the spatial clustering of SEs promoting gene activation. This process might be also important in oncogenic pathways or developmental abnormalities, as RYBP is essential for their maintenance.

## Conclusions

RYBP regulates SE activity. RYBP cooperates with TrxG component to promote the deposition of active histone modifications at SEs, and regulates eRNA transcription on SEs. RYBP generally localizes at SEs in both in vitro cell lines and in vivo tissue-derived cells. Dysfunction of RYBP is associated with various cancers and developmental disorders.

## Electronic supplementary material

Below is the link to the electronic supplementary material.

Supplementary Material 1

Supplementary Material 2

Supplementary Material 3

Supplementary Material 4

## Data Availability

Sequencing data from this study are available at NCBI GEO under accession number GSE261485 and GSE281406. Public datasets utilized in this research are listed in Table S2.

## Abbreviations

ESC: Embryonic stem cell
EPC: Epidermal progenitor
MEC: Mesodermal cell
SE: Super-enhancer
seRNA: enhancer RNA at SE regions
TrxG: Trithorax group
PcG: Polycomb group
PRC1: Polycomb Repressive Complex 1
PRC2: Polycomb Repressive Complex 2
Co-IP: Co-immunoprecipitation
TE: Typical enhancer
ACC: Adrenocortical carcinoma
ESCA: Esophageal carcinoma
KICH: Kidney chromophobe
UCEC: Uterine corpus endometrial carcinoma
UCS: Uterine carcinosarcoma
