# Supplementary material for: Polycomb protein RYBP facilitates super-enhancer activity
Source: Mol Med. 2024 Nov 27;30:236. doi: 10.1186/s10020-024-01006-3 (PMC11603947; doi:10.1186/s10020-024-01006-3)
Supplement: Supplementary file 2 — Supplementary Material 2 [file 10020_2024_1006_MOESM2_ESM.docx]

| **Table S2: Accession number of sequencing data** | | |
| --- | --- | --- |
| **Sample** | **Accession number** | **Publication** |
| ESC, shEV, rep1 | GEO: GSM8145218 | This paper |
| ESC, shEV, rep2 | GEO: GSM8145219 | This paper |
| ESC, sh*Rybp*, rep1 | GEO: GSM8145220 | This paper |
| ESC, sh*Rybp*, rep2 | GEO: GSM8145221 | This paper |
| ESC, sh*Wdr5*, rep1 | GEO: GSM8145222 | This paper |
| ESC, sh*Wdr5*, rep2 | GEO: GSM8145223 | This paper |
| ESC, *Rybp*^+/+^,H3K4me3, rep1 | GEO: GSM8145228 | This paper |
| ESC, *Rybp*^+/+^,H3K4me3, rep2 | GEO: GSM8145229 | This paper |
| ESC, *Rybp*^-/-^,H3K4me3, rep1 | GEO: GSM8145230 | This paper |
| ESC, *Rybp*^-/-^,H3K4me3, rep2 | GEO: GSM8145231 | This paper |
| ESC, *Rybp*^+/+^,WDR5, rep1 | GEO: GSM8145232 | This paper |
| ESC, *Rybp*^+/+^,WDR5, rep2 | GEO: GSM8145233 | This paper |
| ESC, *Rybp*^-/-^WDR5, rep1 | GEO: GSM8145234 | This paper |
| ESC, *Rybp*^-/-^,WDR5, rep2 | GEO: GSM8145235 | This paper |
| ESC, *Rybp*^+/+^,H3K27ac, rep1 | GEO: GSM8145236 | This paper |
| ESC, *Rybp*^+/+^,H3K27ac, rep2 | GEO: GSM8145237 | This paper |
| ESC, *Rybp*^-/-^,H3K27ac, rep1 | GEO: GSM8145238 | This paper |
| ESC, *Rybp*^-/-^,H3K27ac, rep2 | GEO: GSM8145239 | This paper |
| ESC, *Rybp*^+/+^,Input | GEO: GSM8145240 | This paper |
| ESC, *Rybp*^-/-^,Input | GEO: GSM8145241 | This paper |
| RYBP.rat.CM | GEO: GSM8620032 | This paper |
| Input.rat.CM | GEO: GSM8620033 | This paper |
| ChIP.WT.ESC.RYBP.GSE136584.Rep1 | GEO: GSM4052119 | (Zepeda-Martinez et al. 2020) |
| ChIP.WT.ESC..GSE136584.Rep2 | GEO: GSM4052120 | (Zepeda-Martinez et al. 2020) |
| ChIP.RYBP_null.ESC.RYBP.GSE136584.Rep1 | GEO: GSM4052123 | (Zepeda-Martinez et al. 2020) |
| ChIP.RYBP_null.ESC.RYBP.GSE136584.Rep2 | GEO: GSM4052124 | (Zepeda-Martinez et al. 2020) |
| ChIP.WT.ESC.RYBP.PRJNA604675 | GEO: SAMN13978255 | (Zhao et al. 2020) |
| ChIP.RYBP.KO.ESC.RYBP.PRJNA604675 | GEO: SAMN13978256 | (Zhao et al. 2020) |
| ChIP.ESC.RYBP.GSE83135.Rep1 | GEO: GSM2192980 | (Rose et al. 2016) |
| ChIP.ESC.RYBP.GSE83135.Rep2 | GEO: GSM2192981 | (Rose et al. 2016) |
| ChIP.ESC.RYBP.GSE83135.Rep3 | GEO: GSM2192982 | (Rose et al. 2016) |
| ChIP.ESC.RYBP.GSE147919 | GEO: GSM4448898 | (Wei et al. 2022) |
| ChIP.ESC.RYBP.HiChIP.Rep1 | GEO: GSM4448888 | (Wei et al. 2022) |
| ChIP.ESC.RYBP.HiChIP.Rep2 | GEO: GSM4448889 | (Wei et al. 2022) |
| ChIP.ESC.H3K4me1 | GEO: GSM723016 | (Shen et al. 2012) |
| ChIP.ESC.H3K27ac | GEO: GSM851278 | (Shen et al. 2012) |
| ChIP.ESC.MED1 | GEO: GSM560347 | (Kagey et al. 2010) |
| ChIP.ESC.OCT4 | GEO: GSM1082340 | (Whyte et al. 2013) |
| ChIP.ESC.SOX2 | GEO: GSM1082341 | (Whyte et al. 2013) |
| ChIP.ESC.NANOG | GEO: GSM1082342 | (Whyte et al. 2013) |
| ChIP.ESC.RING1B.Rep1 | GEO: GSM2533855 | (Bonev et al. 2017) |
| ChIP.ESC.RING1B.Rep2 | GEO: GSM2533856 | (Bonev et al. 2017) |
| ChIP.ESC.H3K27me3 | GEO: GSM1058999 | (Das et al. 2014) |
| ChIP.ESC.H3K4me3 | GEO: GSM1059001 | (Das et al. 2014) |
| ChIP.ESC.Jarid2 | GEO: GSM465889 | (Peng et al. 2009) |
| ChIP.ESC.Ezh2 | GEO: GSM480161 | (Peng et al. 2009) |
| ChIP.ESC.Cbx7 | GEO: GSM1041373 | (Morey et al. 2013) |
| ChIP.ESC.CBX2 | GEO: GSM2293301 | (Lau et al. 2017) |
| ChIP.ESC.Phc1 | GEO: GSM2393580 | (Kundu et al. 2017) |
| ChIP.ESC.Pol II.GSE20530 | GEO: GSM515667 | (Rahl et al. 2010) |
| ChIP.ESC..pol II.GSE143969 | GEO: GSM4277439 | (Wang et al. 2020) |
| ChIP.ESC..pol II.GSE65491 | GEO: GSM1534642 | (Li et al. 2015) |
| ChIP.ESC..pol II.GSE114655 | GEO: GSM3061012 | (Lynch et al. 2020) |
| ChIP.ESC.pol II.S2P.GSE114655 | GEO: GSM3061006 | (Lynch et al. 2020) |
| ChIP.ESC.pol II.S2P.GSE149999 | GEO: GSM4519339 | (Ding et al. 2021) |
| ChIP.ESC.pol II.S2P.GSE43231 | GEO: [GSM1059014](https://www.ncbi.nlm.nih.gov/geo/query/acc.cgi?acc=GSM1059014) | (Das et al. 2014) |
| ChIP.ESC.pol II.S2P.GSE20530 | GEO: GSM515663 | (Rahl et al. 2010) |
| ChIP.ESC.pol II.S2P.GSE161993.Rep1 | GEO: GSM4929359 | (Fursova et al. 2021) |
| ChIP.ESC.pol II.S2P.GSE161993.Rep2 | GEO: GSM4929360 | (Fursova et al. 2021) |
| ChIP.ESC.pol II.S2P.GSE161993.Rep3 | GEO: GSM4929361 | (Fursova et al. 2021) |
| ChIP.ESC.pol II.S2P.GSE161993.Rep4 | GEO: GSM4929362 | (Fursova et al. 2021) |
| ChIP.ESC.pol II.S5P | GEO: GSM515662 | (Rahl et al. 2010) |
| ChIP.ESC.RBBP5 | GEO: GSM566278 | (Ang et al. 2011) |
| ChIP.mbrain.H3K27ac | GEO: GSM1479204 | (Gao et al. 2014) |
| ChIP.mbrain.RYBP | GEO: GSM5581256 | (Liu et al. 2021) |
| ChIP.EPC.H3K27ac_rep1 | GEO: GSM3069602 | (Cohen et al. 2018) |
| ChIP.EPC.H3K27ac_rep2 | GEO: GSM3069603 | (Cohen et al. 2018) |
| ChIP.EPC.H3K4me1_rep1 | GEO: GSM3069606 | (Cohen et al. 2018) |
| ChIP.EPC.H3K4me1_rep2 | GEO: GSM3069607 | (Cohen et al. 2018) |
| ChIP.EPC.Rybp_rep1 | GEO: GSM3070320 | (Cohen et al. 2018) |
| ChIP.EPC.Rybp_rep2 | GEO: GSM3070321 | (Cohen et al. 2018) |
| ChIP.MEC.RYBP | GEO: GSM1657391 | (Morey et al. 2015) |
| ChIP.MEC.H3K27ac.Rep_1 | GEO: GSM1163098 | (Wamstad et al. 2012) |
| ChIP.MEC.H3K27ac.Rep_2 | GEO: GSM1163099 | (Wamstad et al. 2012) |
| ChIP.MEC.H3K4me3.Rep_1 | GEO: GSM1163117 | (Wamstad et al. 2012) |
| ChIP.MEC.H3K4me3.Rep_2 | GEO: GSM1163118 | (Wamstad et al. 2012) |
| ChIP.MEC.H3K4me1.Rep_1 | GEO: GSM1163087 | (Wamstad et al. 2012) |
| ChIP.MEC.H3K4me1.Rep_2 | GEO: GSM1163088 | (Wamstad et al. 2012) |
| ChIP.Rat.CM.NTC.H3K27ac.rep1 | GEO: SRR27557386 | (Hong et al. 2024) |
| ChIP.Rat.CM.NTC.H3K27ac.rep2 | GEO: SRR27557387 | (Hong et al. 2024) |
| ChIP.Rat.CM.DOX.H3K27ac.rep1 | GEO: SRR27557385 | (Hong et al. 2024) |
| ChIP.Rat.CM.DOX.H3K27ac.rep2 | GEO: SRR27557384 | (Hong et al. 2024) |
| RNA.MEF.rep1 | GEO: GSM470579 | (Heng et al. 2010) |
| RNA.MEF.rep2 | GEO: GSM470580 | (Heng et al. 2010) |
| RNA.reprogramming.day3.rep1 | GEO: GSM470583 | (Heng et al. 2010) |
| RNA.reprogramming.day3.rep2 | GEO: GSM470584 | (Heng et al. 2010) |
| RNA.reprogramming.day3.rep3 | GEO: GSM470585 | (Heng et al. 2010) |
| RNA.reprogramming.day7.rep1 | GEO: GSM470589 | (Heng et al. 2010) |
| RNA.reprogramming.day7.rep2 | GEO: GSM470590 | (Heng et al. 2010) |
| RNA.reprogramming.day7.rep3 | GEO: GSM470591 | (Heng et al. 2010) |
| RNA.reprogramming.day11.rep1 | GEO: GSM470595 | (Heng et al. 2010) |
| RNA.reprogramming.day11.rep2 | GEO: GSM470596 | (Heng et al. 2010) |
| RNA.reprogramming.day11.rep3 | GEO: GSM470597 | (Heng et al. 2010) |
| RNA.iPSC.rep1 | GEO: GSM470571 | (Heng et al. 2010) |
| RNA.iPSC.rep2 | GEO: GSM470572 | (Heng et al. 2010) |
| RNA.ESC.rep1 | GEO: GSM470569 | (Heng et al. 2010) |
| RNA.ESC.rep2 | GEO: GSM470570 | (Heng et al. 2010) |
| RNA.differentiation.mESC.rep1 | GEO: GSM4322468 | Patent: Biomaterials for 3D Cell Growth and Differentiation |
| RNA.differentiation.mESC.rep2 | GEO: GSM4322469 |  |
| RNA.differentiation.mESC.rep3 | GEO: GSM4322470 |  |
| RNA.differentiation.mESC.rep4 | GEO: GSM4322471 |  |
| RNA.differentiation.day9.rep1 | GEO: GSM4322472 |  |
| RNA.differentiation.day9.rep2 | GEO: GSM4322473 |  |
| RNA.differentiation.day9.rep3 | GEO: GSM4322474 |  |
| RNA.differentiation.day9.rep4 | GEO: GSM4322475 |  |
| RNA.differentiation.day14.rep1 | GEO: GSM4322476 |  |
| RNA.differentiation.day14.rep2 | GEO: GSM4322477 |  |
| RNA.differentiation.day14.rep3 | GEO: GSM4322478 |  |
| RNA.differentiation.day14.rep4 | GEO: GSM4322479 |  |

**Reference**

Ang YS, Tsai SY, Lee DF, Monk J, Su J, Ratnakumar K, et al. Wdr5 Mediates Self-Renewal and Reprogramming via the Embryonic Stem Cell Core Transcriptional Network. Cell. 2011;145:183-197.

Bonev B, Cohen NM, Szabo Q, Fritsch L, Papadopoulos GL, Lubling Y, et al. Multiscale 3D Genome Rewiring during Mouse Neural Development. Cell. 2017;171:557-+.

Cohen I, Zhao DJ, Bar C, Valdes VJ, Dauber-Decker KL, Nguyen MB, et al. PRC1 Fine-tunes Gene Repression and Activation to Safeguard Skin Development and Stem Cell Specification. Cell Stem Cell. 2018;22:726-+.

Das PP, Shao Z, Beyaz S, Apostolou E, Pinello L, De Los Angeles A, et al. Distinct and Combinatorial Functions of Jmjd2b/Kdm4b and Jmjd2c/Kdm4c in Mouse Embryonic Stem Cell Identity. Mol Cell. 2014;53:32-48.

Ding L, Paszkowski-Rogacz M, Mircetic J, Chakraborty D, and Buchholz F. The Paf1 complex positively regulates enhancer activity in mouse embryonic stem cells. Life Sci Alliance. 2021;4:

Fursova NA, Turberfield AH, Blackledge NP, Findlater EL, Lastuvkova A, Huseyin MK, Dobrinic P, and Klose RJ. BAP1 constrains pervasive H2AK119ub1 to control the transcriptional potential of the genome. Gene Dev. 2021;35:749-770.

Gao ZH, Lee P, Stafford JM, von Schimmelmann M, Schaefer A, and Reinberg D. An AUTS2-Polycomb complex activates gene expression in the CNS. Nature. 2014;516:349-+.

Heng JCD, Feng B, Han JY, Jiang JM, Kraus P, Ng JH, et al. The Nuclear Receptor Nr5a2 Can Replace Oct4 in the Reprogramming of Murine Somatic Cells to Pluripotent Cells. Cell Stem Cell. 2010;6:167-174.

Hong Y, Li XL, Li J, He QY, Huang MB, Tang YB, Chen X, Chen J, Tang KJ, and Wei C. H3K27ac acts as a molecular switch for doxorubicin-induced activation of cardiotoxic genes. Clinical Epigenetics. 2024;16:

Kagey MH, Newman JJ, Bilodeau S, Zhan Y, Orlando DA, van Berkum NL, et al. Mediator and cohesin connect gene expression and chromatin architecture. Nature. 2010;467:430-435.

Kundu S, Ji F, Sunwoo H, Jain G, Lee JT, Sadreyev RI, Dekker J, and Kingston RE. Polycomb Repressive Complex 1 Generates Discrete Compacted Domains that Change during Differentiation. Mol Cell. 2017;65:432-+.

Lau MS, Schwartz MG, Kundu S, Savol AJ, Wang PI, Marr SK, et al. Mutation of a nucleosome compaction region disrupts Polycomb-mediated axial patterning. Science. 2017;355:

Li MM, Gou HF, Tripathi BK, Huang J, Jiang SL, Dubois W, et al. An Apela RNA-Containing Negative Feedback Loop Regulates p53-Mediated Apoptosis in Embryonic Stem Cells. Cell Stem Cell. 2015;16:669-683.

Liu SX, Aldinger KA, Cheng CV, Kiyama T, Dave M, McNamara HK, et al. NRF1 association with AUTS2-Polycomb mediates specific gene activation in the brain. Mol Cell. 2021;81:4663-+.

Lynch CJ, Bernad R, Martínez-Val A, Shahbazi MN, Nóbrega-Pereira S, Calvo I, et al. Global hyperactivation of enhancers stabilizes human and mouse naive pluripotency through inhibition of CDK8/19 Mediator kinases. Nature Cell Biology. 2020;22:1223-+.

Morey L, Aloia L, Cozzuto L, Benitah SA, and Di Croce L. RYBP and Cbx7 Define Specific Biological Functions of Polycomb Complexes in Mouse Embryonic Stem Cells. Cell Reports. 2013;3:60-69.

Morey L, Santanach A, Blanco E, Aloia L, Nora EP, Bruneau BG, and Di Croce L. Polycomb Regulates Mesoderm Cell Fate-Specification in Embryonic Stem Cells through Activation and Repression Mechanisms. Cell Stem Cell. 2015;17:300-315.

Peng JC, Valouev A, Swigut T, Zhang JM, Zhao YM, Sidow A, and Wysocka J. Jarid2/Jumonji Coordinates Control of PRC2 Enzymatic Activity and Target Gene Occupancy in Pluripotent Cells. Cell. 2009;139:1290-1302.

Rahl PB, Lin CY, Seila AC, Flynn RA, McCuine S, Burge CB, Sharp PA, and Young RA. c-Myc Regulates Transcriptional Pause Release. Cell. 2010;141:432-445.

Rose NR, King HW, Blckledge NP, Fursova NA, Ember KJI, Fischer R, Kessler BM, and Klose RJ. RYBP stimulates PRC1 to shape chromatin-based communication between Polycomb repressive complexes. Elife. 2016;5:

Shen Y, Yue F, McCleary DF, Ye Z, Edsall L, Kuan S, et al. A map of the cis-regulatory sequences in the mouse genome. Nature. 2012;488:116-120.

Wamstad JA, Alexander JM, Truty RM, Shrikumar A, Li FG, Eilertson KE, et al. Dynamic and Coordinated Epigenetic Regulation of Developmental Transitions in the Cardiac Lineage. Cell. 2012;151:206-220.

Wang XL, Gerber A, Chen WY, and Roeder RG. Functions of paralogous RNA polymerase III subunits POLR3G and POLR3GL in mouse development. P Natl Acad Sci USA. 2020;117:15702-15711.

Wei C, Jia LM, Huang XN, Tan J, Wang ML, Niu J, et al. CTCF organizes inter-A compartment interactions through RYBP-dependent phase separation. Cell Research. 2022;32:744-760.

Whyte WA, Orlando DA, Hnisz D, Abraham BJ, Lin CY, Kagey MH, Rahl PB, Lee TI, and Young RA. Master Transcription Factors and Mediator Establish Super-Enhancers at Key Cell Identity Genes. Cell. 2013;153:307-319.

Zepeda-Martinez JA, Pribitzer C, Wang J, Bsteh D, Golumbeanu S, Zhao Q, et al. Parallel PRC2/cPRC1 and vPRC1 pathways silence lineage-specific genes and maintain self-renewal in mouse embryonic stem cells. Sci Adv. 2020;6:eaax5692.

Zhao J, Wang M, Chang L, Yu J, Song A, Liu C, et al. RYBP/YAF2-PRC1 complexes and histone H1-dependent chromatin compaction mediate propagation of H2AK119ub1 during cell division. Nat Cell Biol. 2020;22:439-452.
