# Supplementary material for: Polycomb protein RYBP facilitates super-enhancer activity
Source: Mol Med. 2024 Nov 27;30:236. doi: 10.1186/s10020-024-01006-3 (PMC11603947; doi:10.1186/s10020-024-01006-3)
Supplement: Supplementary file 4 — Supplementary Material 4 [file 10020_2024_1006_MOESM4_ESM.docx]

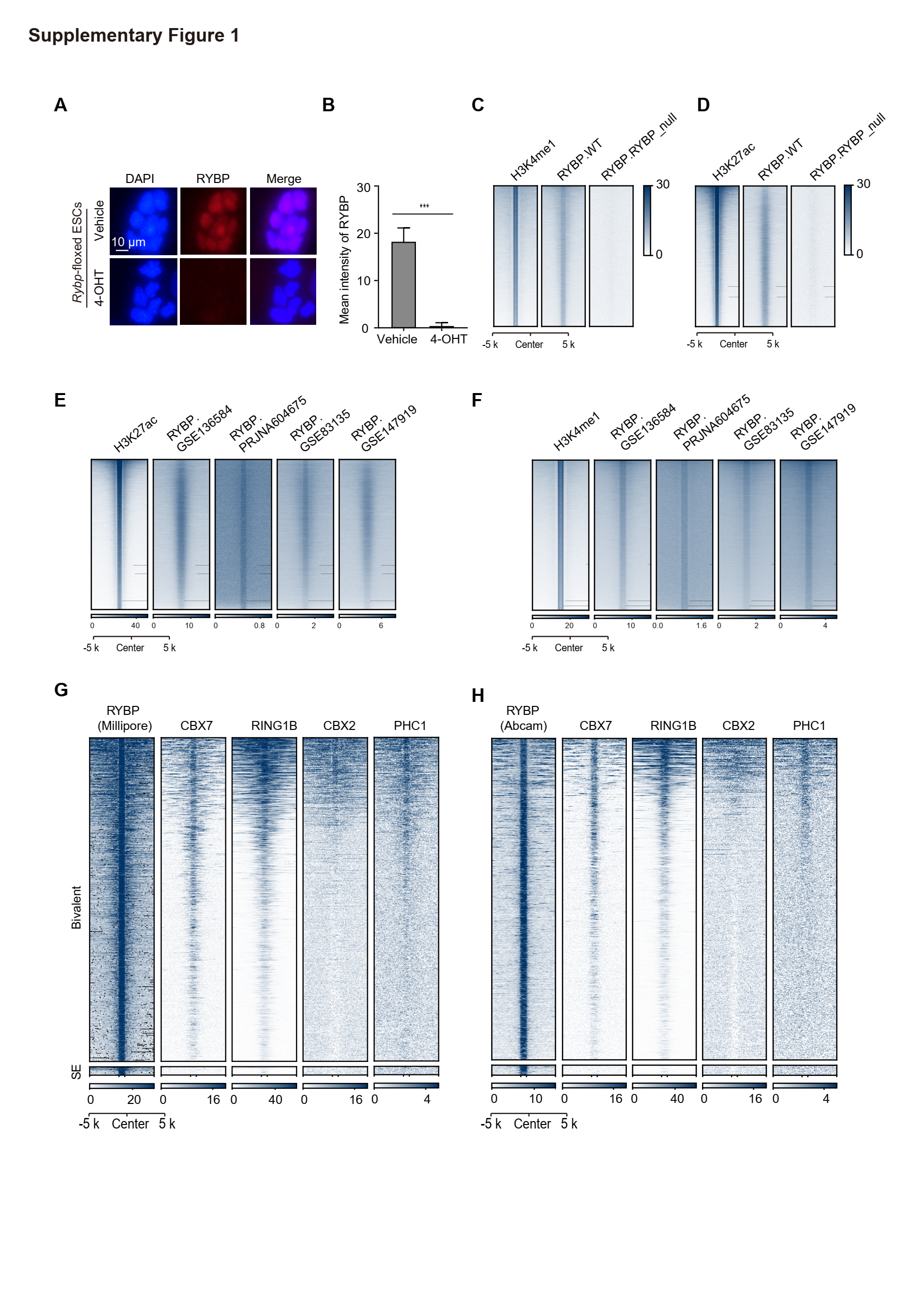


**Fig. S1** RYBP occupies super-enhancers in embryonic stem cells. (**A-B**) Immunofluorescence images (**A**) and quantification (**B**) showing the RYBP signal in WT and RYBP KO ESCs, 94 and 156 cells were detected for vehicle and 4-OHT group, respectively. Two-tailed Welch’s t-test.  (**C-D**) RYBP signals from WT or RYBP_null ESC at RYBP_H3K4me1 (**C**) or RYBP_H3K27ac (**D**) co-binding loci. (**E-F**) RYBP signals from four datasets at H3K27ac and H3K4me1 loci. (**G-H**) In bivalent or SE regions, the signal of Polycomb proteins at RYBP loci defined from different antibodies (Millipore or Abcam).


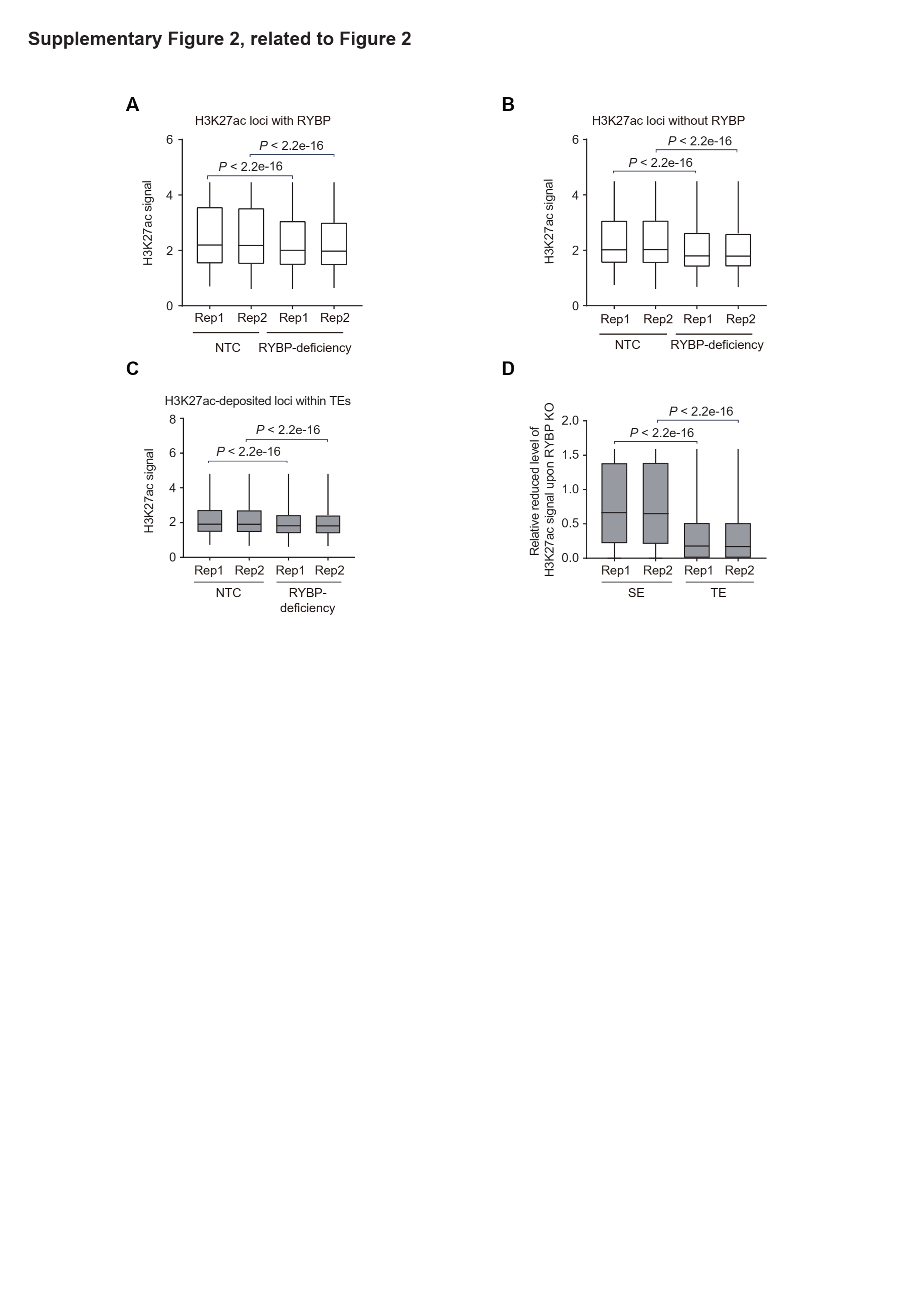


**Fig. S2** RYBP depletion impairs the enrichment of H3K27ac at SEs. (**A-B**) At SEs, boxplot showing the H3K27ac signal at H3K27ac loci with (**A**) or without (**B**) RYBP after RYBP-depletion, one-tailed Wilcoxon test, 2 replicates for the two groups. (**C**) Boxplot showing the H3K27ac signal at TEs after RYBP-depletion, one-tailed Wilcoxon test, 2 replicates for the two groups. (**D**) Relative reduced level of H3K27ac signal upon RYBP KO at SEs and TEs, one-tailed Wilcoxon test, 2 replicates for the two groups.


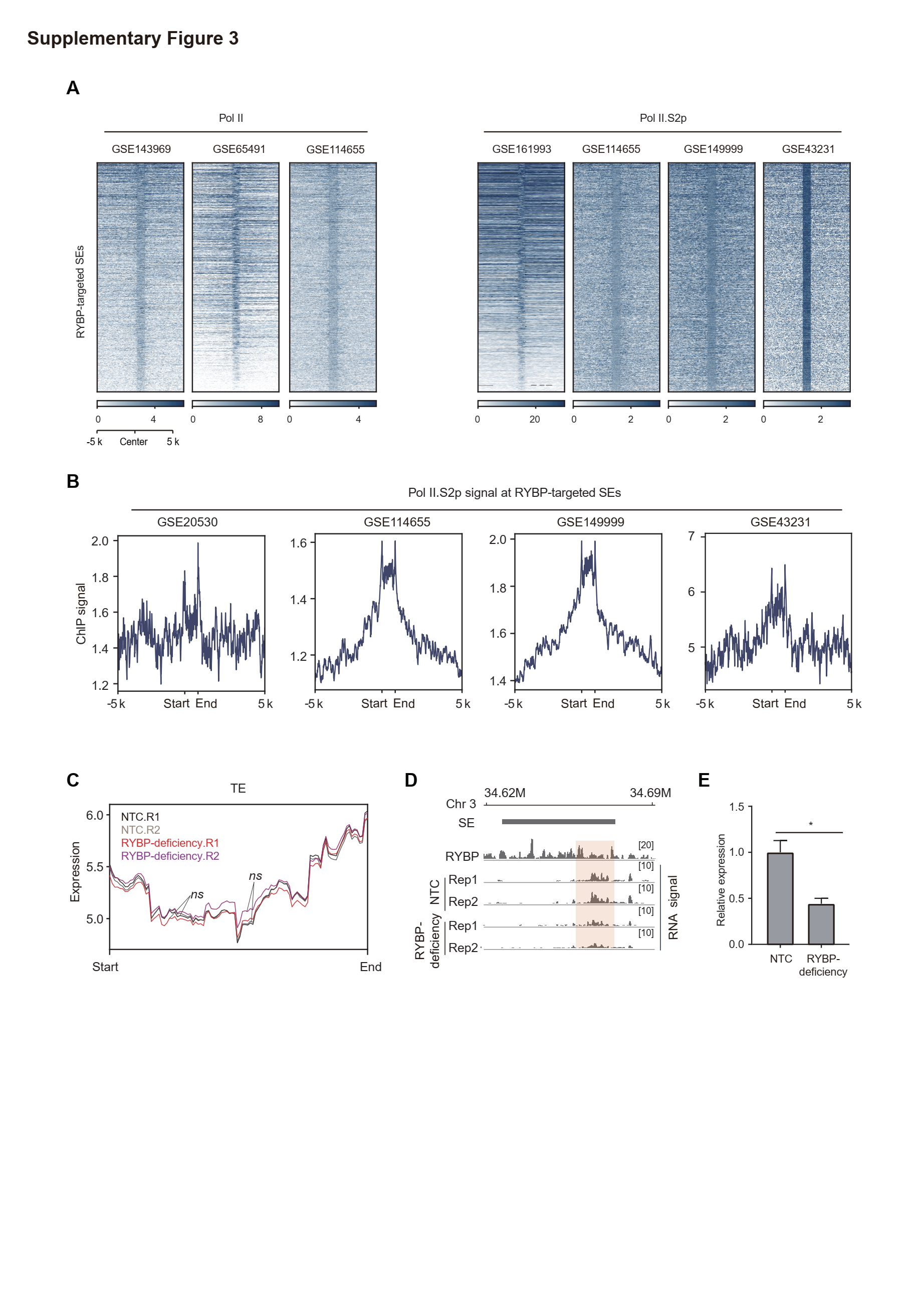


**Fig. S3** RYBP depletion reduces the expression of seRNAs. (**A-B**) Heatmap showing the ChIP-seq signal of Pol II and Pol II.S2p from different datasets. (**C**) Quantification showing the RNA expression level at SEs before and after RYBP deficiency, one-tailed K-S test, 2 replicates for the two groups, which shows that the RNA expression in NTC groups did not significantly higher than that in RYBP-deficiency group. (**D-E**) Representative locus showing the RNA expression level at SEs before and after RYBP deficiency, one-tailed Welch’s t-test, 2 replicates for the two groups.


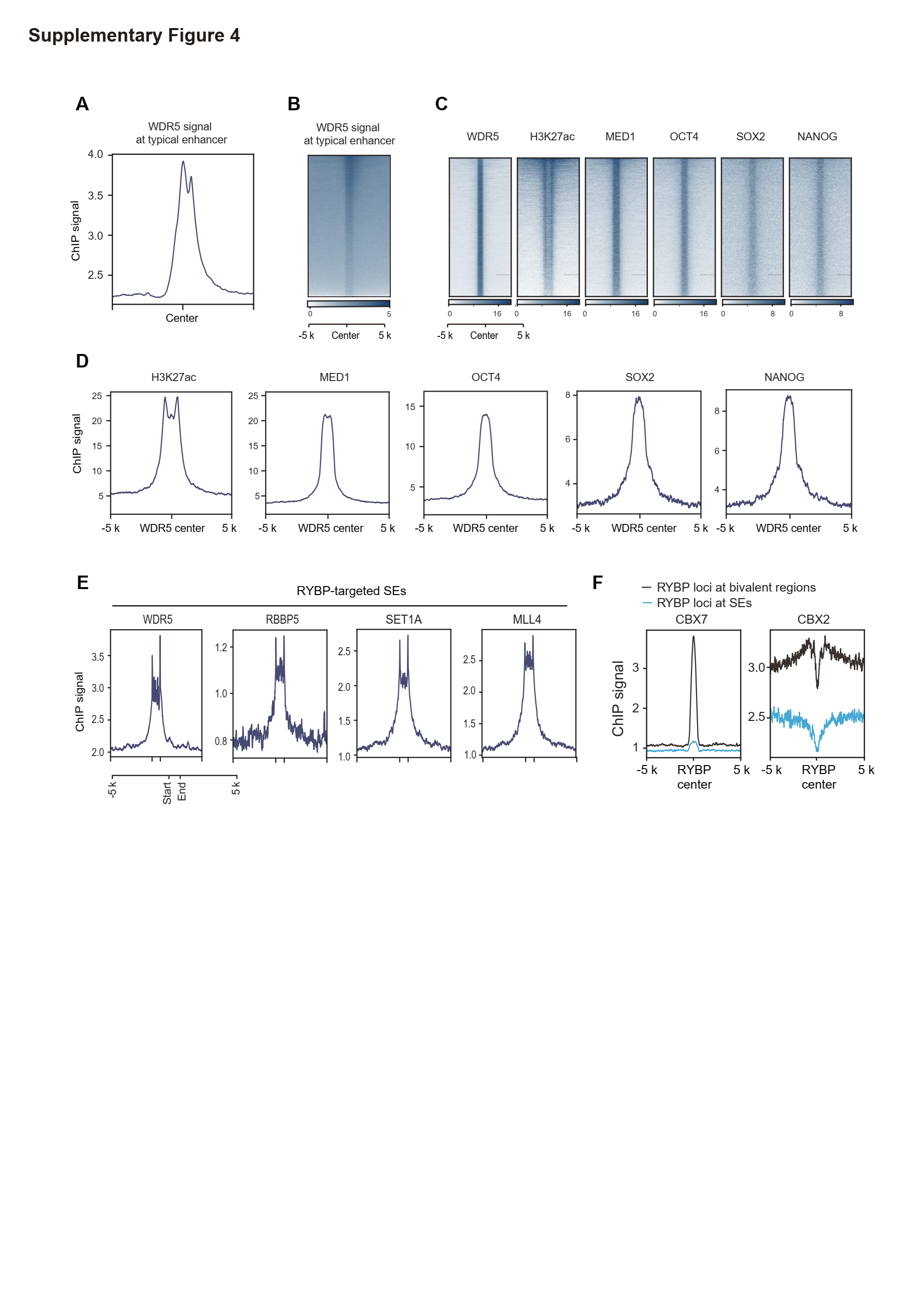


**Fig. S4** RYBP co-localizes with TrxG components at SEs. (**A-B**) WDR5 signal at typical enhancers. (**C-D**) Heatmap showing the deposition of H3K27ac, MED1, OCT4, SOX2 and NANOG at WDR5 loci. (**E**) ChIP-seq signal of TrxG components at RYBP-targeted SEs. (**F**) ChIP-seq signal of PcG components at the RYBP peak center.


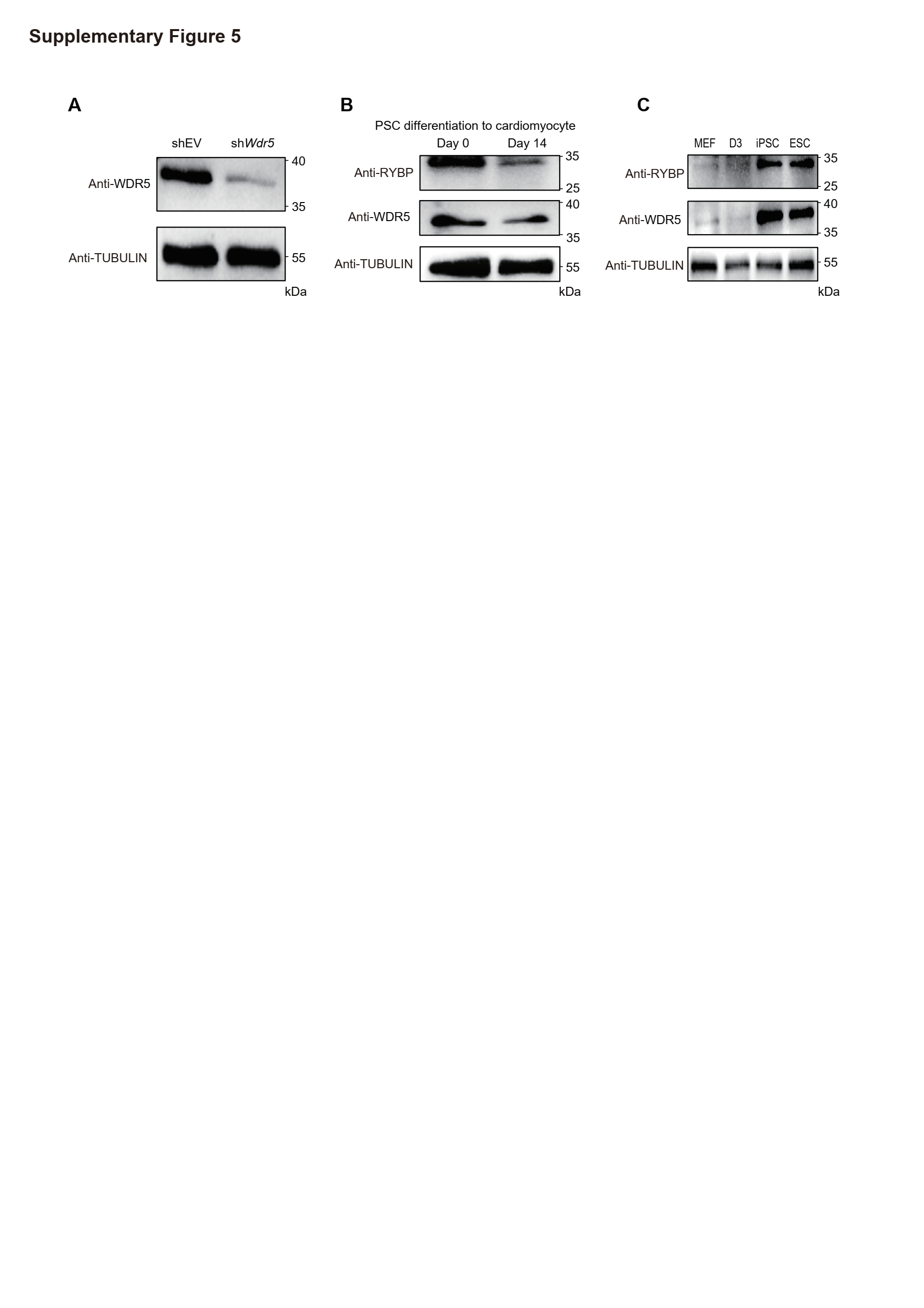


**Fig. S5** Deficiency of TrxG component reduces the expression of RYBP-regulated seRNAs. (**A**) Western blot showing the expression of WDR5 protein in WDR5 knockdown ESCs. (**B-C**) Western blot showing the expression of RYBP and WDR5 proteins during PSC differentiation to cardiomyocyte and reprogramming.


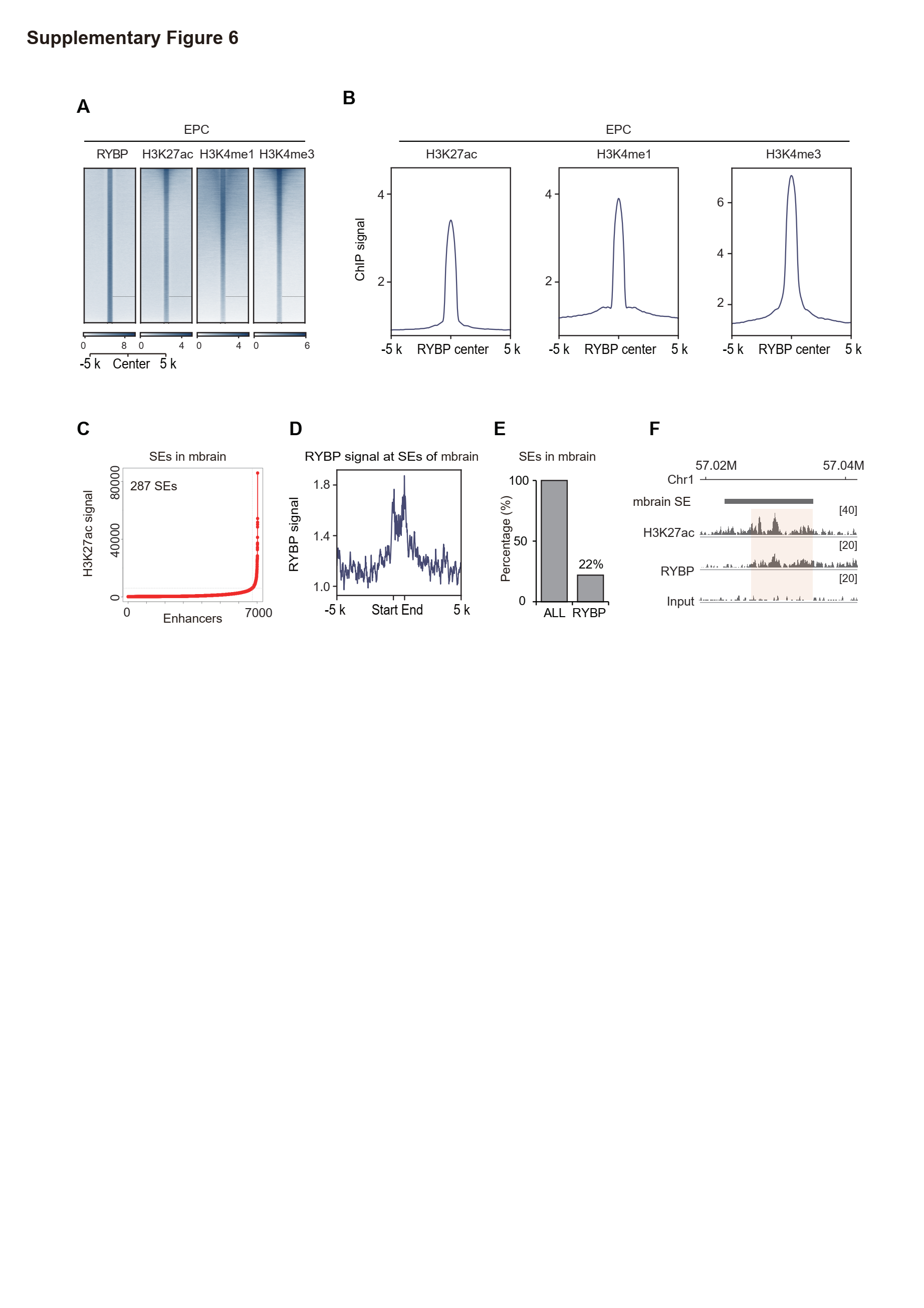


**Fig. S6** RYBP generally occupies SEs across cell types and species. (**A-B**) H3K27ac, H3K4me1 and H3K4me3 signal at RYBP loci in EPCs. (**C**) H3K27ac signal at enhancers in mbrain-derived cells. (**D**) RYBP signal at SEs in mbrain-derived cells. (**E**) The percentage of RYBP-targeted SEs among all SEs in mbrain-derived cells. (**F**) The representative loci showing the deposition of RYBP at SE in mbrain-derived cells.


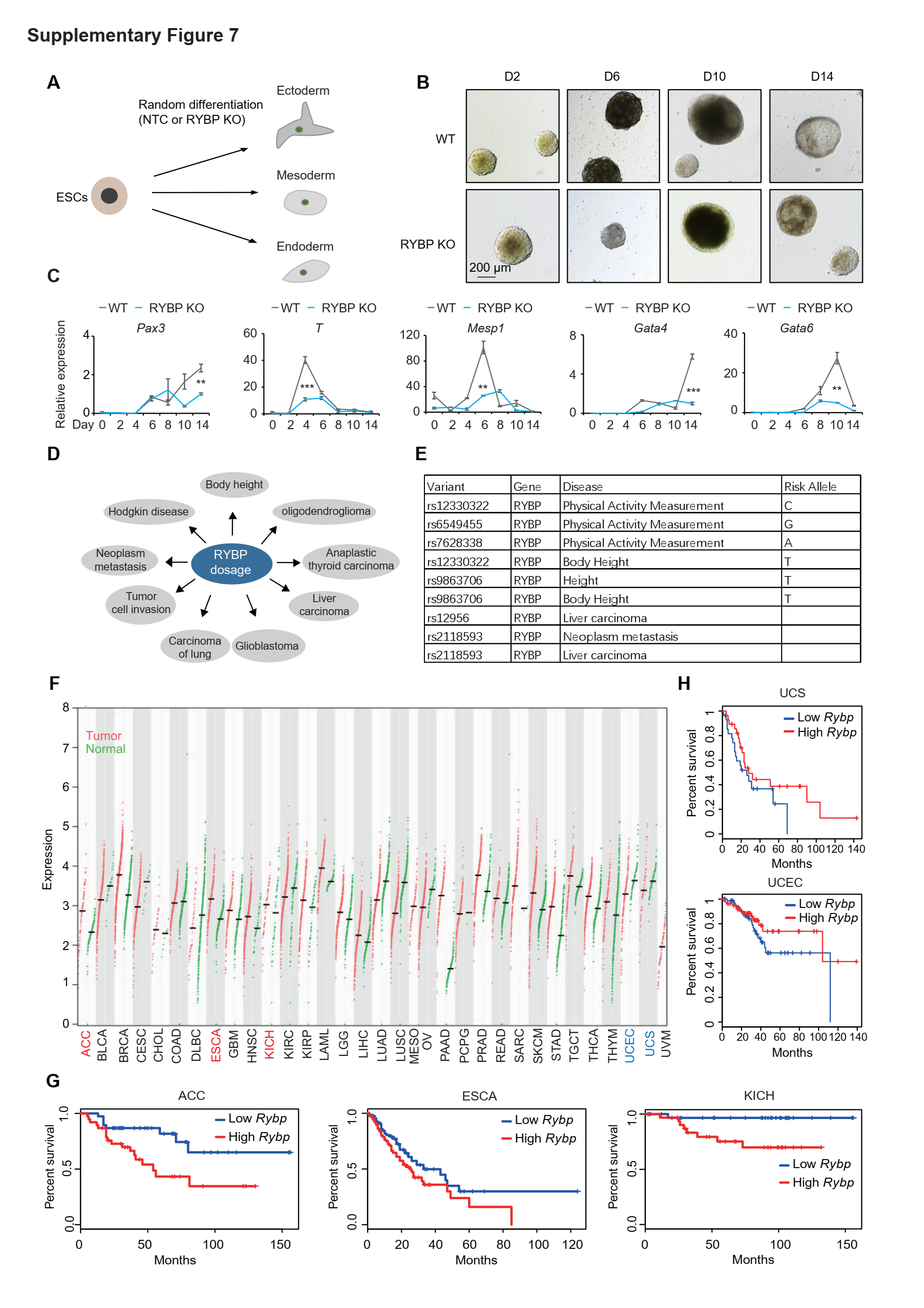


**Fig. S7** RYBP is required for cell fate control. (**A**) Schematic of the experimental design for investigating the role of RYBP in ESC differentiation. (**B**) Representative images showing the EBs in different days. (**C**) RT-qPCR detects the expression of genes in EBs from WT or RYBP KO ESCs, two-tailed Welch’s t-test, 3 replicates for each group. (**D-E**) A summary of diseases associated with the abnormal dosage and mutation of RYBP. (**F**) Relative expression of RYBP in normal individuals compared to tumor patients. (**G-H**) The survival curve displays the relationship between RYBP dosage and the survival time of different cancer patients, including Adrenocortical carcinoma (ACC), Esophageal carcinoma (ESCA), Kidney Chromophobe (KICH), uterine carcinosarcoma (UCS) and Uterine Corpus Endometrial Carcinoma (UCEC).
